# A spin-photon interface in the telecom C-band with long hole spin dephasing time

**DOI:** 10.1038/s41467-026-77282-w

**Published:** 2026-09-02

**Authors:** Johannes M. Michl, Reza Hekmati, Mohamed Helal, Giora Peniakov, Yorick Reum, Jochen Kaupp, Quirin Buchinger, Jaewon Kim, Andreas T. Pfenning, Yong-Hoon Cho, Sven Höfling, Tobias Huber-Loyola

**Affiliations:** 1 Julius-Maximilians-Universität Würzburg, Physikalisches Institut and Würzburg-Dresden Cluster of Excellence ctd.qmat, Lehrstuhl für Technische Physik, Würzburg, Am Hubland Deutschland; 2 Department of Physics, Korea Advanced Institute of Science and Technology (KAIST), Daejeon, Republic of Korea; 3 Karlsruhe Institute of Technology, Institute of Photonics and Quantum Electronics (IPQ) and Center for Integrated Quantum Science and Technology (IQST), Karlsruhe, Germany

**Keywords:** Quantum physics, Quantum dots, Single photons and quantum effects

## Abstract

Matter qubits that maintain coherence over extended timescales are essential for many pursued applications in quantum communication and quantum computing. Significant progress has already been made on extending coherence times of spins in semiconductor quantum dots while interfacing them with photons in the near-infrared wavelength range. However, similar results for quantum dots emitting at the telecom range, crucial for many applications, have so far lagged behind. Here, we report on InAs/InAlGaAs quantum dots integrated in a deterministically placed circular Bragg grating emitting at 1.55 μm. We quantify the g-factors of electrons and holes from polarization-resolved measurements of a positive trion in an in-plane magnetic field and study the dynamics of the ground-state hole spin qubit by heralding the spin in two-photon correlation measurements. We quantify the inhomogeneous dephasing times for the electron and hole for various magnetic fields and find a value of ${T}_{2}^{*}=(21.8\pm 0.2)$ ns for the hole spin, for an optimal magnetic field of *B *= 20 mT.

## Abstract (web summary)

Spin-photon interfaces based on telecom wavelength quantum dots require long-lived spin qubits for quantum networking applications. Here, the authors demonstrate a hole spin dephasing time exceeding 20 ns in a telecom band quantum dot integrated in a circular Bragg grating, setting a new benchmark for spin coherence in this platform.

## Introduction

The phenomenon of quantum superposition is nowadays most prominently featured in the concept of qubits—the basic building blocks of quantum information applications like quantum computing and quantum communication. While the term flying qubit almost exclusively refers to photons ^1^, stationary qubits can be realized in systems such as trapped ions^2,3^, superconducting circuits^4,5^, nitrogen-vacancy (NV) centers in diamond^6^ or semiconductor quantum dots (QDs)^7^.

An interface between flying and stationary qubits allows for local operations on the stationary qubits and quantum information transfer between them using the flying qubits. In solid-state, interfacing stationary qubits with photons is possible due to spin-dependent optical transitions—as can for instance be found in NV centers or QDs—that connect the polarization of an emitted photon with the state of a confined spin^8^. This connection leads to non-classical correlations which most notably manifest in the entanglement between a solid-state spin and a photon^9–11^. Based thereon, a work by Lindner and Rudolph from 2009^12^ proposed to utilize the spin-qubit as an entangler, enabling the generation of a linear cluster state of entangled photons. These one-dimensional entangled states are highly sought-after, since they can be fused to high-order graph states^13^ which, in turn, are the resource states for measurement-based quantum computing^14^ as well as for all-photonic quantum repeaters^15,16^.

A linear cluster state from a quantum dot was first demonstrated in 2016^17^ in the near-infrared (NIR) range below 1 μm and has since then been repeatedly reported for NIR quantum dots^18,19^, also featuring indistinguishable photons^20,21^. Despite this progress, the largest cluster states achieved to date come from neutral atoms^13,22^ in cavities, as their isolation from the environment allows for coherence times of >1 ms. In solid-state emitters, by contrast, the interaction with the environment is limiting the coherence times, although through nuclear spin cooling^23,24^ and advanced decoupling protocols^25,26^ values >100 μs are attainable. Yet these coherence-enhancing methods have not been combined with cluster-state generation, leaving quantum-dot implementations effectively constrained by the inhomogeneous dephasing time $${T}_{2}^{*}$$ of the spin qubit. There, state-of-the-art values are obtained in the NIR-range, reaching up to $${T}_{2}^{*} > $$ 70 ns for a hole spin^27^.

At the same time, practical considerations for long-distance quantum communication and integration with silicon photonics motivate a shift toward emission in the telecom C-band at (1.53–1.56) μm, where optical-fiber losses are minimal. Although NIR-range photons can be frequency-shifted via down-conversion^11,28,29^, the process is intrinsically probabilistic—conversion succeeds only with finite probability, typically introducing additional timing jitter—and is furthermore limited in efficiency, making it difficult to reach the thresholds required for scalable architectures and quantum advantage. Thus, while NIR quantum dots currently offer the most favorable spin-coherence properties, telecom-band operation remains the regime of highest relevance for scalable quantum-network applications.

Substantial improvements in the optical quality of telecom-emitting quantum dots have been achieved through refined growth techniques that optimize material properties. In recent works, we have reported on the enhancing effect of e.g., ternary digital alloying and the optimization of the Ostwald-ripening time on photon properties^30,31^. Furthermore, much progress has been made on the integration of telecom QDs into circular Bragg-gratings (CBGs), leading to Purcell-enhanced single photon emission with high brightness and short radiative lifetimes^32^, as well as indistinguishability^31,33,34^. Full coherent control of a QD spin qubit emitting telecom photons has been achieved only a few years back^35^ followed by spin-photon entanglement measurements^36^, both featuring modest spin coherence times <1 ns. Only recently, slightly higher values ≤7 ns could be reported utilizing quasi-resonant excitation schemes^37–40^. Until now, studies of telecom-emitting quantum dots hosting spins with inhomogeneous dephasing times long enough to support large photonic cluster-state generation have been absent.

Here, we report on measurements of a hole spin from an InAs/InAlGaAs quantum dot integrated into a circular Bragg grating with a ${T}_{2}^{*}$ exceeding 21 ns. The quantum dots studied here, in combination with a tailored CBG design for enhanced emission around 1.5 μm, have shown excellent optical qualities, including new state-of-the-art values of two-photon interference visibilities that exceed >90%^31,41^. We utilize p-shell quasi-resonant excitation and exploit the polarization memory of a positive trion (*X*^+^) to study the excited-state spin evolution as well as the coherence dynamics of a ground-state hole-qubit.

## Results

The data we report on here focus on the optical transition of a positively charged excitonic complex *X*^+^, also referred to as a positive trion. Basic characterization and trion identification were conducted by performing power- and polarization series (cf. supplementary section S1.2). We measured InAs/InAlGaAs QDs grown by molecular beam epitaxy, which were integrated into a circular Bragg grating resonator using hyperspectral imaging and deterministic placement. This material platform and resonator design combined have shown to be a source of indistinguishable single photons with reduced lifetime due to the Purcell enhancement^30–32^. The deterministic placement of the photonic nanostructure ensures that the QD couples well to the cavity mode and that the polarization bias imprinted by the CBG for displaced emitters is minimized^42,43^.

### Zeeman splitting and lifetime oscillations

**Fig. 1:**
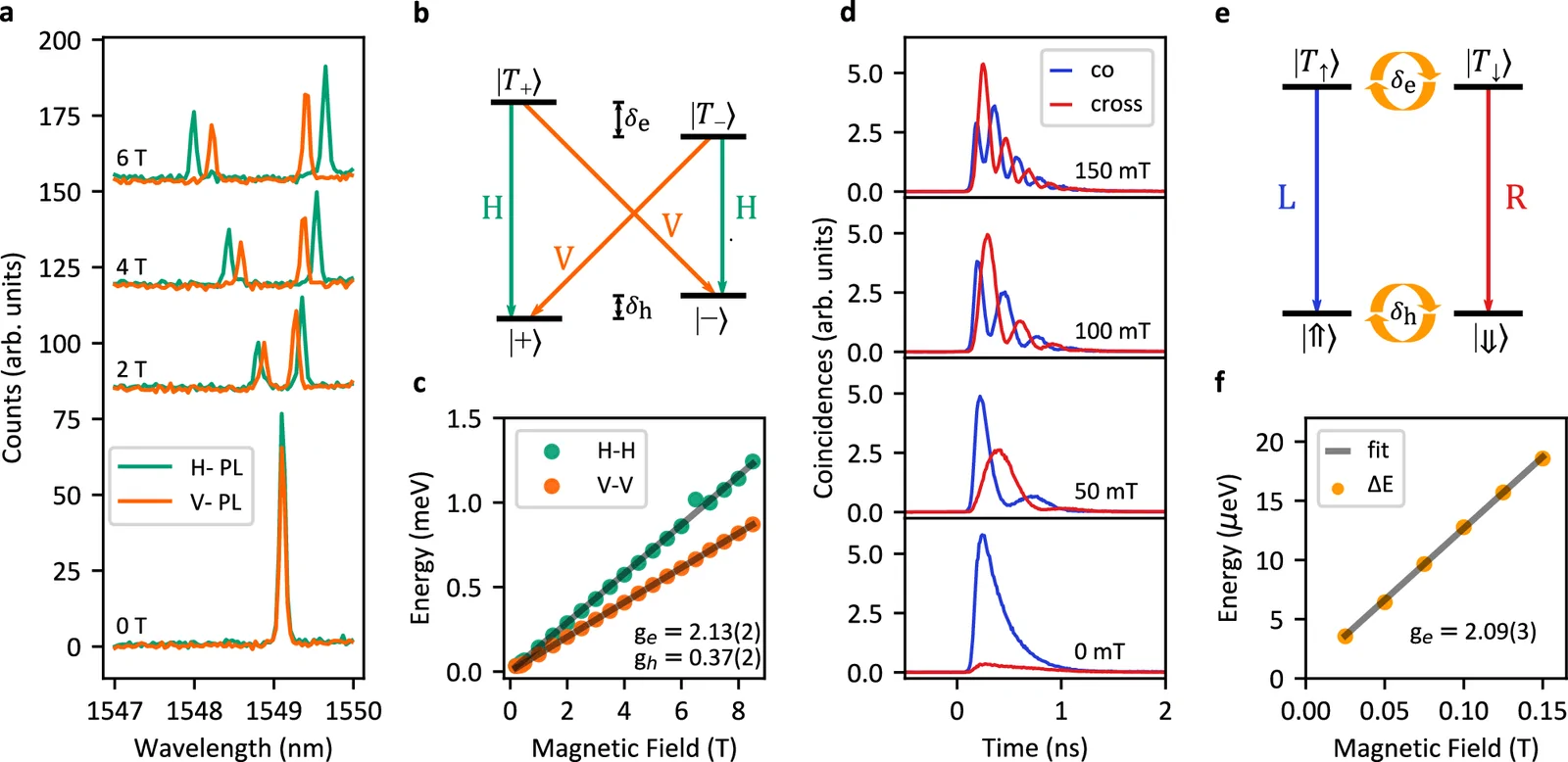
Splittings and g-factors from polarizations resolved measurements in a magnetic field. **a** Spectral line filtered in H- and V-polarization. The lines split for increasing magnetic field, with a stronger splitting observed for the H-component. **b** Sketch of the trion energy level scheme. When the splitting is sufficiently large, the optical transitions are governed by linearly (H/V) polarized photon emission. **c** Energy splitting between H (green)- and V (orange) polarized emission lines. From the splitting, the g-factors of the excited and ground level spins can be calculated. **d** Time- and polarization-resolved trace of the *T*^+^ emission at zero magnetic field and for small fields *B*_*x*_ ≤ 0.15 T. For 0 T, a polarization memory of 0.865 ± 0.001 can be extracted. For *B*_*x*_ > 0 T, the exponential decays of the trion lifetime traces are modulated by oscillations that stem from spin precessions. The frequency is proportional to the induced energy splitting of the excited state, as illustrated in (**e**). **f** Linear fit to the observed energy splitting. From this, a g-factor of 2.09 ± 0.03 can be calculated.

We analyzed the Zeeman splitting of a positive trion in the presence of an in-plane magnetic field. At 0 T, the *X*^+^ transition shows no resolvable splitting for orthogonal linear polarizations *H* and *V*, as is depicted in Fig. 1a. It can further be observed that, when ramping up the magnetic field, the trion splits into four distinct lines. The energy of each spectral line marks a transition between eigenstates of the double-*Λ* system formed by the *X*^+^ and the hole *h* in the ground state, as sketched in Fig. 1b. The splitting *δ*_*h*_ of the hole eigenstates $\vert+\rangle$, $\vert -\rangle$ is characterized by the hole g-factor *g*_*h*_, while the splitting *δ*_*e*_ of the trion eigenstates $\vert {T}_{+}\rangle$, $\vert {T}_{-}\rangle$ is governed by the g-factor *g*_*e*_ of the unpaired electron.

We extracted the g-factor of the electron and hole by analyzing the Zeeman splitting shifts of the polarization resolved spectral peaks. The energy separations between the two *H*- and *V*-polarized transitions, Δ*H* and Δ*V*, were fitted linearly as a function of the magnetic field as shown in Fig. 1c. From these fits, we obtained *g*_*e*,*x*_ = 2.13 ± 0.02 and *g*_*h*,*x*_ = 0.37 ± 0.02 following Δ*E*_*i*_ = *μ*_*B*_*  g*_*i*,*x*_ Δ*B*_*x*_ with *μ*_*B*_ being the Bohr magneton. Consistent with previous work on (InAs/InGaAs) QDs with a comparable host material and strain^38^ we assign the larger g-factor to the unpaired electron in the trion state, while the smaller value corresponds to the hole in the ground state.

In the absence of a magnetic field, the emission lines from a trion are degenerate in energy according to Kramers’ theorem and thus not spectrally distinguishable. Furthermore, the following selection rules along the $\hat{z}$ direction apply:

1 $$\vert {T}_{\uparrow {/}\downarrow }\rangle \mapsto \vert \Uparrow {/}\Downarrow \rangle \vert L/R \rangle ,$$

with the trion states $\vert {T}_{\uparrow /\downarrow }\rangle$, ground state hole spin states $\vert \Uparrow /\Downarrow \rangle$ and photon polarizations states $\vert L/R\rangle$. This mapping between spin states and photon polarization states leads to an experimentally observable polarization memory effect: Exciting the trion (quasi-) resonantly with *R* (*L*) circularly polarized light is, upon recombination, followed by the emission of a photon with the same polarization *R* (*L*).

Here, we applied a quasi-resonant excitation scheme that begins with a single hole, denoted $\vert h\rangle$, confined in the QD. Upon optical injection of an electron-hole pair, the system is excited to the higher-energy trion state $\vert {T}^{*}\rangle$ from where it transitions to the trion ground level $\vert T\rangle$ via phonon-assisted relaxation. This scheme has been shown to enable writing of a spin qubit^38^ and was furthermore used for cluster state generation in the NIR range^20^. We selected a resonance by performing a photoluminescence-excitation (PLE) measurement. For this, a tunable cw-laser was scanned from (1465–1540) nm and a resonance at ~1521 nm was chosen (cf. supplement section S1.3). Figure 1(d, bottom panel) depicts a measurement at 0 T from which we could infer a degree of circular polarization (DOCP) of (86.5 ± 0.1)%. The residual deviation from the ideal DOCP contrast is attributed to the p-shell excitation scheme: excited hole states can exhibit material-dependent shell structure and enhanced valence-band mixing, in particular heavy-hole–light-hole admixture^44,45^, which may relax the optical selection rules and open spin-non-conserving relaxation pathways before population of the s-shell; this interpretation is supported by the LA-phonon-assisted excitation control measurement shown in SI Fig. S7. Applying LA-phonon assisted or resonant excitation with the presented DOCP sheme is possible, but will not reveal different physics of the spin dynamics. Throughout the rest of this work, we utilize the polarization memory to initialize the trion in a state $\vert {T}_{\uparrow }\rangle$ ($\vert {T}_{\downarrow }\rangle$) using *L* (*R*)-polarized laser excitation.

The splitting of the trion energy levels in a weak magnetic field (*B*_*x*_ ≤ 150 mT) is not strong enough to separate the emission lines spectrally. In this regime, the effect of the magnetic field is as follows: Since $\vert {T}_{\uparrow }\rangle$ and $\vert {T}_{\downarrow }\rangle$ are both superpositions of the trion eigenstates $\vert {T}_{+}\rangle$ and $\vert {T}_{-}\rangle$ and *B*_*x*_ induces a splitting *δ*_*e*_ = *μ*_*B*_*g*_*e*_*B*_*x*_ of those states, a state $\vert {T}_{\uparrow }\rangle$ will evolve to the state $\vert {T}_{\downarrow }\rangle$ in a time *T* = *h*/2*δ*_*e*_, with *h* being Planck’s constant (cf. Fig. 1e).

This relation becomes plainly visible when measuring the time trace of the trion with excitation and detection in the *R*/*L* polarization basis. Figure 1d depicts the time trace for magnetic fields ranging from 0 T to 150 mT. For non-zero magnetic fields, the exponential decay is modulated by oscillations. We fitted the time traces to extract the oscillation frequencies and converted them into energies. The resulting Δ*E*(*B*) dataset is displayed in Fig. 1f. This enabled us to determine the g-factor from the slope of a linear fit to *g*_*e*_ = 2.09 ± 0.03. Within a 2*σ* interval, this value is in agreement with *g*_*e*_ obtained above for higher magnetic fields.

This leaves the remaining hole g-factor, *g*_*h*_, to govern the splitting of the ground-state eigenstates. Analogous to the case of the excited state, a hole spin $\vert \Uparrow \rangle$ precesses in time around the eigenstate axis. Our observation of this precession is discussed in the following sections.

### Ground state spin precessions

In contrast to the measurements presented in the previous section, the ground-state spin dynamics can not be directly monitored via the time trace of the emitted photon. Here, we made use of the selection rules along the $\hat{z}$-direction again. By performing two-photon correlation experiments we could herald the ground state spin in either $\vert \Downarrow \rangle$ or $\vert \Uparrow \rangle$ by detecting a *R*- or *L*-polarized photon and thus study the evolution of the state before detection of the second photon. In a first experiment we studied the spin under continuous-wave quasi-resonant excitation as has been demonstrated in recent years by various groups^46,47^. Following this, we extend the results by following an approach that utilizes excitation with two pulses with variable delay and polarization.

#### Autocorrelation measurement with continuous excitation

**Fig. 2:**
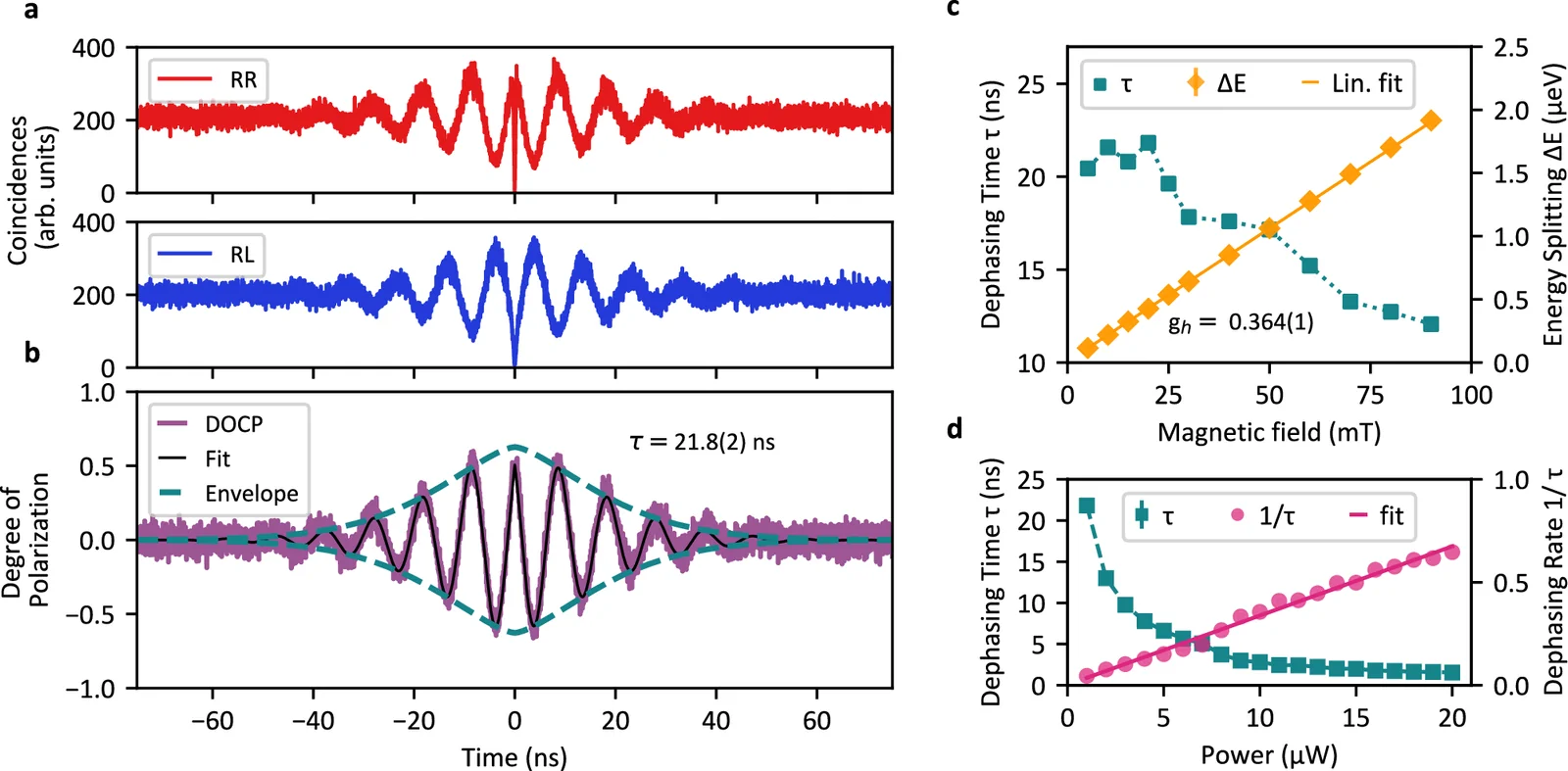
Polarization resolved autocorrelation measurement. The trion was excited continuously at different magnetic fields and with varying laser powers. The excitation polarization was set to *H*, and the detection polarizations were set to either RR (channel 1 channel 2) or RL, enabling the observation of ground state spin precessions. **a** RR (top, red) and RL (bottom, blue) time traces for 1 μW and *B* = 20 mT. Calculating (*RR* − *RL*)/(*RR* + *RL*) yields the DOCP displayed in (**b**). The decaying envelope used to modulate the fitted cosine function yields a coherence time of *τ* = (21.8 ± 0.2) ns. **c** Oscillation frequency / energy splitting extracted at various magnetic fields. From the energy splittings, the g-factor can be determined to 0.364 ± 0.001. Additionally, one can observe a decrease in *τ* for increasing magnetic fields, whereas for low fields *B* ≤ 20 mT a saturation point is reached. **d** Laser-power dependence of the extracted hole-spin dephasing time (${T}_{2}^{*}$) (left axis). The corresponding dephasing rate, $1/{T}_{2}^{*}$, is shown on the right axis. The dephasing rate exhibits an approximately linear dependence on excitation power over the investigated range, consistent with an additional excitation-induced contribution to the measured spin dephasing.

We performed the measurements utilizing a Hanbury Brown and Twiss setup configuration, with cw-excitation and polarization projection of the photons in individually controllable detection channels after a beam splitter. For the data shown in Fig. 2, we used linearly *H*-polarized excitation in order to minimize possible artifacts from optically induced dynamic nuclear polarization. A comparison with circularly polarized excitation is provided in Supplementary Fig. S10. Figure 2a displays the autocorrelation data measured in parallel (top, *RR*) and orthogonal (bottom, *RL*) detection polarization configuration (channel 1 channel 2), with a laser power of 1 μW and an in-plane magnetic field of 20 mT. The excitation polarization was set to *H* in order to provide equal excitation probability of the ground state hole independent of its current spin orientation. Detecting a *R*-polarized photon in channel 1 heralds the spin in its $\vert \Downarrow \rangle$ state. If fast re-excitation occurs there is a high probability for the trion to be in the $\vert {T}_{\downarrow }\rangle$ state, due to the spin preserving quasi-resonant *H* excitation. On the other hand, if the hole spin precesses half a period (*T*_*h*_/2) to the $\vert \Uparrow \rangle$ state before re-excitation the trion state is initialized in $\vert {T}_{\uparrow }\rangle$ which makes emission of a *L* polarized photon at that time much more likely. This coupling of the emitted polarization with the orientation of the hole spin during excitation causes a *π*-shift between the observed oscillations in *RR* and *RL* configuration. Figure 2b displays the DOCP, calculated according to (*RR* − *RL*)/(*RR* + *RL*) from the data of panel (a) and fitted by a combination of cosine function $cos(2\pi {ft}+\phi)$ and stretched decaying envelope term ${e}^{-{(t/\tau )}^{\alpha }}$. This enabled us to extract a dephasing time of *τ* = (21.8 ± 0.2) ns for the ground state spin precession.

We studied the dependence of the fit parameters *f*, *τ* and *α* on both the magnitude of the magnetic field and the excitation power. Parallel to the excited state evolution described in the previous section, the frequency *f* is governed by the g-factor and follows $f = \delta_h/h$ with *δ*_*h*_ = *μ*_*B*_*g*_*h*_*B*_*x*_. Figure 2c features a linear fit to Δ*E*(*B*). The g-factor resulting from the slope of the fit amounts to 0.364 ± 0.001, which is consistent with *g*_*h*_ = 0.37 ± 0.02 measured earlier within one standard deviation. This confirms that the observed signal undulations stem from the ground state spin precessions, as opposed to the oscillations in the lifetime measurements that could be attributed to *g*_*e*_. We would like to draw the reader's attention towards the magnetic field-dependent dephasing time *τ*(*B*), which is also included in Fig. 2c. While increasing *B*_*x*_ leads to an increased level splitting and oscillation frequency, the extracted coherence gets clearly reduced. However, we could identify a maximum value at ${B}_{x}^{opt}=20\,{{\rm{mT}}}$. For lower fields, the extracted dephasing time is slightly reduced. We believe that this effect is coupled with the emergence of a slowly oscillating component in the Fourier transformed signal with frequencies ∣*f*∣ ≤ 10 MHz that starts to dominate the DOCP oscillation for *B*_*x*_ ≤ 10 mT (cf. supplement section S3.1 for more details). For the DOCP fit, this component is filtered out.

A similar dependence of the extracted ${T}_{2}^{*}$ on the excitation power is observed. As depicted in Fig. 2d, increasing the excitation power gradually reduces the hole-spin dephasing time. The corresponding dephasing rate $1/{T}_{2}^{*}$, shown on the right axis, exhibits an approximately linear dependence on excitation power over the investigated range. This behavior is consistent with an additional excitation-induced decoherence channel. A possible microscopic origin is an additional excitation event occurring before completion of the free spin evolution, effectively shortening the available ground-state evolution time.

For both studies presented in Fig. 2, *α* was fitted as a free parameter and found to exhibit values ranging from ~1.5 to ~0.5 (cf. supplementary material). These values deviate from the Gaussian envelope (*α* = 2) expected for dephasing dominated solely by a quasi-static Gaussian Overhauser-field distribution, as is the case for electrons when the dephasing is dominated by frozen nuclear-field fluctuations. Since the Fermi-contact hyperfine interaction is strongly suppressed for hole spins, we interpret the extracted *α* values as an indication that additional channels, such as non-contact hyperfine interaction, charge-noise-induced *g*-factor fluctuations, and excitation-induced re-excitation, contribute to the observed decay envelope^48–50^. The extrapolation of *τ* towards even smaller laser powers shows that the measured coherence of ~22 ns could be exceeded for an unperturbed system. Therefore, we studied the hole spin under pulsed excitation, which we elaborate on in the next section.

#### Probing the spin with pulses

We studied the spin evolution of the ground-state hole using two-photon correlations (2PC). For this measurement, the laser pulse was split into two paths, with the delay in one arm controlled by a motorized linear stage. The delayed pulse is hereafter referred to as pulse 2, whereas the other pulse is pulse 1. For this measurement, we used two separate excitation paths, as well as two detection channels (CH1 and CH2). The detection channels are synchronized to the laser trigger that is created by pulse 1. Both the excitation polarization and the detection polarization projection can be controlled individually for each pulse and detection channel. Thus, we can excite and project each photon in an arbitrary basis. The in-plane magnetic field was set to *B*_*x*_ = 150 mT.

**Fig. 3:**
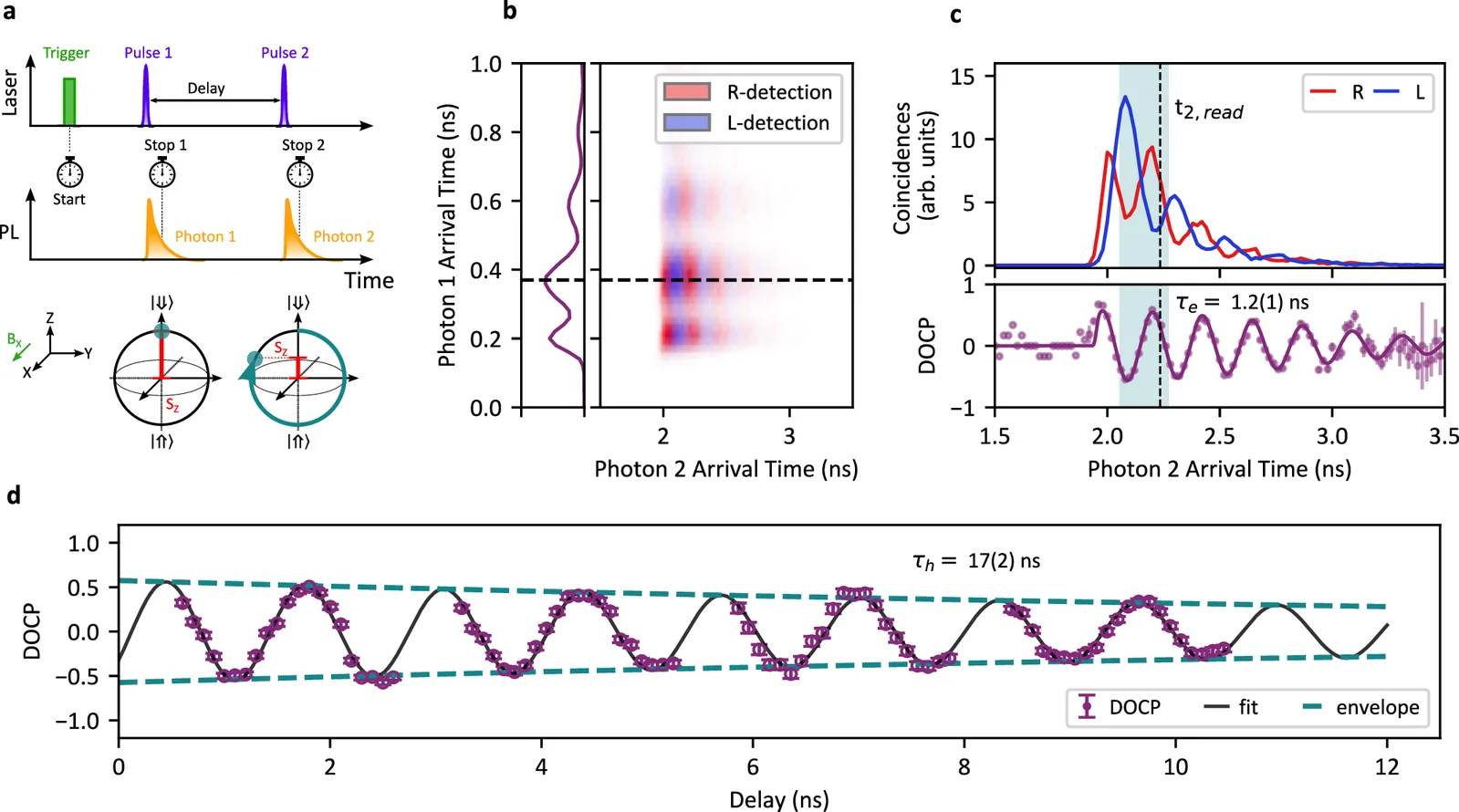
Pulsed coherence time measurement. The hole spin is probed utilizing a two-photon correlation experiment. **a** Schematic illustration of the measurement sequence. The experiment is synchronized by an electronic trigger signal from the laser, which starts the measurement clock. The emitted laser pulse is split into two pulses, and the second pulse arrives delayed due to an optical path of controllable length. The polarization for both pulses can be controlled individually. The same applies to the projection of the resulting photons, whose arrival in two separate channels, CH1 and CH2, stops the clock. Detecting the first photon in *R* (after *R*-excitation) heralds the spin in $\left\vert \Downarrow \right\rangle$. After undisturbed precession with the Larmor frequency around an in-plane magnetic field of 150 mT, the hole spin is probed with an *H*-polarized laser pulse and projected to both *R* and *L*. **b** Resulting two-dimensional time trace of photon 2 for a pulse delay of 1.6 ns. The data of both circular polarizations is superimposed in a colormap. The time traces are analyzed along the horizontal direction with regard to a fixed arrival time of the CH1 photon at *t*_*X*,*CH*1_ = 370 ps (cf. dashed black line). The resulting one-dimensional time traces are displayed in (**c**). Top panel: Correlated 〈*RR*〉 (red) and 〈*RL*〉 (blue) signals. A phase shift of *π* can be observed between the oscillating time traces. Bottom panel: DOCP resulting from the datasets in the top panel with a fit that yields a lower bound for the electron spin dephasing time of *τ*_*e *_≥ (1.2 ± 0.1) ns. A vertical dashed line marks the point of readout, which is varied over a window of 220 ps (shaded area). **d** DOCP at *t*_2,*read*_(Δ*t*). The resulting oscillation reflects the ground-state spin-precession of the hole. From a fit, a precession frequency of (763 ± 1) MHz and a dephasing time of *τ*_*h*_ = (17.0 ± 2.2) ns are extracted.

Figure 3a illustrates the measurement protocol, which is described in detail below. After excitation by pulse 1 (*R*-polarization) and subsequent detection of a *R*-polarized photon in CH1, the spin is heralded in $\vert \Downarrow \rangle$ according to $\vert {T}_{\downarrow }\rangle \longmapsto \vert \Downarrow \rangle \vert R\rangle$. Following this, the hole spins precesses freely around the eigenstate axis in the time Δ*t* between the two pulses with a precession time of *T*_*h*_(150 mT) = 1.36 ns. The *H*-polarized pulse 2 then transfers the spin superposition to the excited state while maintaining its phase. For several delays Δ*t* = (0.61-10.45) ns, a pair of two datasets was acquired, projecting the second photon in CH2 to *R* and *L*, respectively. This enabled us to determine the DOCP after Δ*t* to:

2 $$DOCP={S}_{z}=\frac{\langle RR\rangle -\langle RL\rangle }{\langle RR\rangle+\langle RL\rangle },$$

with *S*_*z*_ being the Stokes parameter in the *Z*-basis. Figure 3b maps two overlaid datasets of the second photon arriving in CH2, projected to *R* and *L* respectively. Here, the delay of the two laser pulses was set to 1.6 ns. When being summed up and projected on the vertical axis (detection of first photon in CH1), the data show the fingerprint of the oscillating lifetime trace (cf. Fig. 1d). We analyzed a horizontal cut through the data at *t*_*CH*1_ = 370 ps as indicated by the black dashed line. This slice of data is plotted in Fig. 3(c, top) and features counter-oscillating *R*/*L* time traces. We would like to highlight that without correlating the two photon detection events, exciting with *H* would not result in an oscillating lifetime. The fact that we observe oscillations in the lifetime here shows that we can effectively initialize the spin with the projection of the first photon and probe it with the second laser pulse. In Fig. 3(c, bottom), we plot and fit the DOCP that results from the top panels' *R* and *L* time traces. The fit reveals a sudden rise in the DOCP as the photon 2 arrives, followed by a dampened oscillation which does not fully vanish before the trion decays and the oscillation is lost in noise. Our fit therefore yields a lower bound of *τ*_*e*_ ≥ (1.2 ± 0.1) ns for the electron spin dephasing time that governs the evolution of the trion state.

We proceed by extracting the DOCP for different delays in the following way: We chose an arbitrary time *t*_2_ after the excitation time of the second photon *t*_*pulse*2_, for which we read out *S*_*z*_(*t*_2,*read*_) with *t*_2,*read*_ = *t*_*pulse*2_ + *t*_2_. Figure 3d shows the extracted DOCP with respect to the pulse delay for *t*_2_ = 230 ps (cf. dashed vertical line in Fig. 3c). We fit the data according to

3 $$A(t)=C+{A}_{0}\exp (-{(t/{T}_{2}^{*})}^{\alpha })\cos (\omega t+\varphi ),$$

with *ω* = 2*πf*. For the pulsed measurement, we fix *α* = 1 to avoid over-parameterization of the fit; this choice is used to extract a characteristic free-induction decay time and is not intended as a microscopic assumption on the underlying noise spectrum. This yields a spin dephasing time of ${T}_{2}^{*}(230\,\mbox{ps})=(17.0\pm 2.2)$ ns. Since the fitted value of ${T}_{2}^{*}$ varies slightly with the readout point due to noise on the data, we considered the average over a phase of 2*π* of the excited state precession, see shaded area in Fig. 3d and supplement Fig. S13. This yields a mean dephasing time of ${\overline{T_{2}^{*}}}=(15.9\pm 1.7)$ ns. Similarly, the precession frequency is averaged over this window to $\bar{f}=(0.761\pm 0.002)$ GHz, which calculates to a g-factor of 0.362 ± 0.001, in agreement with the value *g*_2_ found earlier in this work (cf. Fig. 1).

## Discussion

We proceed by comparing the g-factors measured here with other works on InAs/InAlGaAs QDs grown on an InP substrate. Extensive studies by Belykh et al. on ensemble emission of nominally similar dots using pump-probe spectroscopy have found electron g-factors ~1.9 and hole g-factors ~0.6, with huge out-of-plane and small in-plane anisotropy^51,52^. Compared to these values, the single QD investigated here shows a larger difference ∣*g*_*e*_ − *g*_*h*_∣ with *g*_*e*_ ≃ 2.15 and *g*_*h*_ ≃ 0.35, both deviating from the above-mentioned values by 0.25. This deviation is significantly larger than in-plane g-factor spreads of the QD ensemble determined by Belykh et al. to Δ*g* < 0.1 and therefore probably originates from a slightly different dot shape leading to different confinement, In-Ga intermixing or strain. We interpret our results to strengthen our identification of the studied line to be a positive trion, with the excited state spin dynamics governed by the electron and the ground-state single-spin precession determined by the hole.

In addition, this identification is in agreement with the long coherence time of the precessing hole described in this work. Studies on 900 nm emitting dots conducted via pump-probe experiments and in the absence of an external magnetic field have revealed an electron spin depolarization time of ~2 ns^53^. Similar reports comparing electron and hole spins have measured one order of magnitude larger hole spin dephasing times of ~20 ns, which can be attributed to a weaker hyperfine interaction of the heavy-hole with surrounding nuclei^54^. The ${T}_{2}^{*}$ values we could extract from our measurements mark a major step towards closing the gap between NIR- and telecom QDs, with record values still residing in the NIR-range^27^. The presence of indium is generally expected to enhance hyperfine-induced spin dephasing compared with indium-free III–V quantum dots, due to the large nuclear spin of indium (*I* = 9/2) and its sizable hyperfine coupling^39^. However, this argument applies most directly to electron spins, for which the Fermi-contact hyperfine interaction is the dominant coupling to the nuclear spin bath. For hole spins, the Fermi-contact term is strongly suppressed because of the predominantly *p*-like symmetry of the valence-band Bloch functions. Therefore, the hole-spin coherence is not expected to be limited by the same hyperfine mechanism as in the electron-spin case. Instead, the relevant dephasing channels include non-contact hyperfine interaction, heavy-hole–light-hole mixing, and electric-field-induced fluctuations of the hole *g*-factor^50,55^. Consequently, the presence of indium does not by itself preclude observing a measurable hole-spin coherence time in telecom quantum dots. Importantly, Evers et al.^56^ have recently demonstrated coherent hole-spin dynamics in ensembles of self-assembled InAs/InAlGaAs quantum dots emitting at telecom wavelengths, i.e., in the same material platform considered here, showing that indium-containing telecom quantum dots can support measurable hole-spin coherence despite the presence of indium nuclei. The actual ${T}_{2}^{*}$ is nevertheless expected to depend on several material- and device-specific parameters, including alloy composition, strain, dot size, wave-function localization, heavy-hole–light-hole admixture, and the local charge-noise environment. Further improvements should therefore be possible through charge-stabilized diode structures, optimized growth conditions to reduce alloy and charge disorder, and band-structure engineering aimed at minimizing heavy-hole–light-hole mixing and *g*-factor sensitivity to electric-field noise.

Another point of interest that our results entail is the approach for measuring ${T}_{2}^{*}$ displayed in Fig. 3. We could probe the unperturbed evolution of our hole spin between two laser pulses by heralding the spin during detection of the first photon. This protocol can, in principle, be combined with preparation sequences applied before the heralding step, provided that the interval between heralding and readout remains a free-evolution interval. Such pre-pulses may change the role of the heralding photon from initialization to verification/post-selection, or modify the nuclear-spin bath and thus the apparent ${T}_{2}^{*}$^23,24^; however, sequences involving additional spin rotations or dynamical decoupling would measure a protocol-dependent coherence time rather than the free-induction ${T}_{2}^{*}$ extracted here^25,27^. While measurements that are conceptually similar, like continuous excitation or evaluating oscillations in the trion lifetime trace can also quantify ${T}_{2}^{*}$, they fall short in different ways: The first case drives the $\vert \Downarrow \rangle \mapsto \vert{T}_{\downarrow }\rangle$ transition continuously which shortens the measured coherence time exponentially with applied laser power (see our results in Fig. 2). The latter case was used by this and other groups^37,38^ in order to provide a lower boundary for the excited state spin coherence, but is ultimately limited by the radiative lifetime of the trion as has become apparent for the value of *τ*_*e*_ = (1.2 ± 0.1) ns we could extract from the pulsed correlation measurement for the electron spin. Many additional studies have measured ${T}_{2}^{*}$ using Ramsey-interference. In contrast to Ramsey interferometry, the present approach extracts ${T}_{2}^{*}$ directly from polarization-resolved photon correlations and does not require coherent spin-control pulses or calibration of *π*/2 rotations, since the spin is initialized by heralding. Ramsey interferometry remains the standard technique for coherent spin control, whereas our method provides a complementary approach for characterizing free spin evolution through the spin-photon interface.

A further advantage of the present approach is that it can be applied directly in the weak-field regime relevant for polarization-encoded spin-photon protocols. This is important because high-field Ramsey measurements do not necessarily provide the most representative benchmark for this regime: for magnetic fields ${B}_{x}^{ext} > 4\,{{\rm{T}}}$, the spin dephasing time has been shown to decrease inversely with the external magnetic field, following ${T}_{2}^{*}\propto 1/{B}_{x}^{ext}$^27^ consistent with magnetic-field-enhanced sensitivity to fluctuations of the spin splitting. Our measurements at low in-plane magnetic fields show a similar qualitative trend of reduced extracted dephasing time with increasing field, further motivating a characterization method that operates under the same field conditions as the intended spin-photon interface. Since one major application for coherent spin qubits is the generation of linear cluster states, and since for this protocol the in-plane magnetic field is usually in the (10–500) mT range when using polarization encoding, we believe that it is best to quantify ${T}_{2}^{*}$ at similar magnetic fields. In the case of this work, the optimal magnetic field was found to be ${B}_{x}^{opt}=20\,{{\rm{mT}}}$. The downward trend of the dephasing time for increasing magnetic fields that the extracted values follow in Fig. 2c indicates a dephasing time <10 ns likely to be measured at *B*_*x*_ = 150 mT. However, we conducted our pulsed approach using exactly this field, which was due to technical limitations. To resolve multiple precession periods *T*_*h*_ of the hole spin at 20 mT—with *T*_*h*_ ~ 10 ns for this field - pulse picking and optimized pulse delay control are necessary. Still, combining both results suggests that the real inhomogeneous dephasing time for an unperturbed hole spin lies even beyond 30 ns in our sample. We plan to conduct additional measurements in the future to investigate this further.

We have studied a device consisting of an InAs/InAlGaAs quantum dot integrated in a deterministically placed circular Bragg grating. We have identified a positive trion in a polarization-resolved magnetic field series under above-bandgap illumination and have been able to determine the g-factors for electrons and holes in the QD to *g*_*e*_ = 2.134 ± 0.016 and *g*_*h*_ = 0.367 ± 0.016. Utilizing a p-shell resonance found in a PLE measurement, we have found a polarization memory of (86.5 ± 0.1)% and have studied the ps-resolved time evolutions of excited- and ground state spin of the trion in a weak magnetic field. From the observed oscillation frequencies, we have been able to confirm the earlier found g-factor values. Furthermore, we have quantified the dephasing time of the ground-state hole in second-order autocorrelation measurements using continuous-wave excitation. We have explored various magnetic fields and have found an optimal field of 20 mT at which we measured a hole dephasing time of (21.8 ± 0.2) ns, which, to the best of our knowledge, is the longest dephasing time measured for an electron or hole spin in telecom-emitting quantum dots so far.

Motivated by our additional finding that the excitation power of the continuous-wave laser strongly affects the measured dephasing, we have tested a new approach that combines the established spin heralding technique with a probing of the ground-state spin using ultrashort optical pulses after free evolution. There, we have found a dephasing time of (15.9 ± 1.7) ns for a magnetic field of 150 mT, which is consistent with our previous findings and suggests that applying the pulsed approach at 20 mT may result in even higher values of ${T}_{2}^{*}$. We plan to investigate this in future work.

The positive trion studied in our device exhibits long ground-state spin coherence and short excited-state lifetime due to cavity integration. The combination of these features makes it an ideal candidate for studying more complex spin manipulation protocols like dynamical decoupling or for extending to three-photon correlations.

## Methods

### QD growth

The InAs quantum dots (QDs) were grown by gas-source molecular beam epitaxy (MBE) on an InP substrate to achieve emission in the Telecom C-band. The heterostructure consists of a 226 nm InAlGaAs membrane lattice-matched to InP, serving as the optical cavity layer. Below the membrane, an InGaAs etch-stop layer was incorporated. The QDs were formed via Stranski-Krastanov growth by depositing a few monolayers of InAs under elevated arsenic flux, followed by a brief ripening step and capping with the surrounding InAlGaAs barrier. Digital alloying of the quaternary layers was employed to enhance material homogeneity and control strain. The full growth procedure and structural characterization are detailed in previous work, see Refs. ^30–32^.

### CBG design

The circular Bragg grating (CBG) cavities were optimized using three-dimensional finite-difference time-domain (FDTD) simulations to achieve strong Purcell enhancement at the targeted Telecom C-band wavelengths. The design parameters, including center disk radius, grating period, and gap width, were systematically varied to maximize the cavity quality factor and minimize the mode volume while maintaining efficient vertical out-coupling. The resulting geometry supports a Gaussian far-field profile and robust performance against fabrication deviations, following the design principles established in previous works^30–32^.

For fabrication, the optimized CBG pattern was defined by high-resolution electron-beam lithography and transferred into the InAlGaAs membrane by inductively coupled plasma etching using an Ar/Cl_2_ chemistry. Prior to this, a 480 nm dielectric AlOx layer was deposited by sputtering to form the bottom spacer layer, followed by an evaporated Au mirror providing high reflectivity and vertical mode confinement. The sample was then bonded upside-down onto a GaAs carrier substrate using a benzocyclobutene (BCB) adhesive in a flip-chip process, ensuring thermo-mechanical stability. The original InP substrate and buffer layers were subsequently removed by selective wet etching, exposing the InAlGaAs membrane containing the quantum dots. The final device stack thus comprises the suspended InAlGaAs membrane with integrated QDs, the sputtered AlOx layer, and the gold mirror, forming a high-performance CBG cavity suitable for deterministic QD coupling.

### Deterministic CBG placement

In order to center the optimized CBG as well as possible around a preselected QD, we used a hyperspectral imaging technique combined with two image photoluminescence imaging.

As a first step, alignment markers with a periodic marker design were deposited on the sample surface, dividing it into different fields of 37 μm by 37 μm. An excitation laser was focused in the back-focal plane of the objective, thus illuminating the whole field homogeneously. A 4-f setup in combination with a Czerny-Turner spectrometer (slit closed to 50 μm) was used to analyze a strip of the real space image spectrally. Moving /Scanning the lens in front of the spectrometer sideways shifts the image of the sample with respect to the entrance slit, thus changing the analyzed strip. This yields the hyperspectral information of the whole field. From the resulting 3D data cube, suitable QDs were selected for further processing.

To provide the most accurate spatial information, a second camera was utilized together with LED illumination. In the photoluminescence imaging step, two frames were acquired immediately after each other: one with laser illumination and no LED and one frame vice versa. Overlaying both frames shows the QD emission (without spectral information) with respect to the periodic markers. From a fit of both features, the position of the QD with respect to the markers could be determined and translated into coordinates for CBG fabrication by the EBL system. For more detailed information, see Buchinger et al.^43^.

### Excitation lasers

For the experiments presented in this work, three different lasers were used. For the polarization-resolved measurements displayed in Fig. 1a–c a laser diode with an emission wavelength of 1064 nm was used. The PLE scans as well as results of Fig. 2 were obtained through a continuously tunable, fiber coupled cw-laser (Toptica^*TD*^ CTL 1500). The laser pulses required for the results presented in Figs. 1d, e and 3 were produced by a wavelength-tunable, picosecond OPO-laser with 80 MHz repetition rate (APE picoEmerald^*TD*^).

In order to produce two pulses with an adjustable delay, we divided the laser with a fiber beam splitter into two paths. The first path remains in fiber and is guided directly to the setup. The second path is coupled into free space and routed to a dedicated delay setup. There, a motorized linear translation stage with a travel range of 100 mm controls the optical path length before coupling the laser pulse back into fiber. To extend the adjustable pulse delay, the beam is reflected multiple times from the translation stage.

### Polarization control and calibration

We use both liquid-crystal variable retarders (LCVRs) as well as half-wave plates (HWPs) and quarter-wave plates (QWPs) to translate the excitation and detection polarization from the laboratory frame to the QD-frame and vice versa. The detection polarization is especially affected by skewing since the emitted PL is first coupled into fiber and then projected in a dedicated setup on the optical table.

We calibrated the detection polarization through the spectral and temporal signature of the trion as follows: The magnetic field was increased until the emitted PL was distributed between four clearly separable lines (at ~4 T). The polarization was then rotated such that detecting *H* yields the highest- and lowest-energy lines, whereas detecting *V* yields the two middle lines with lower splitting *V*−*V* compared to the *H*−*H* splitting (cf. Fig. 1a–c). In the next step, we reduced the magnetic field to ~100 mT and optimized the visibility of the trion time trace oscillations (cf. Fig. 1d). To do so, we rotated the polarization frame around the *H*/*V* basis until the contrast of the oscillations was maximized (minimized) in the *R*/*L* (*D*/*A*) basis (cf. supplement Fig. S5). The procedure described in this paragraph was repeated iteratively until the best possible calibration was achieved.

The calibration of the excitation polarization follows a similar routine: We used the oscillating lifetime in a weak magnetic field to optimize on the contrast in the *R*/*L* basis; excitation in every other basis (*H*/*V* or *D*/*A*) should result in a flat time trace. The right *H*/*V* basis for the excitation was found through polarization-resolved PLE measurements in high magnetic fields.

## Supplementary information

Supplementary Information

Transparent Peer Review file

## Source data

Source Data Figure 1

Source Data Figure 2

Source Data Figure 3

## Data Availability

The data presented in Figs. 1–3 are available as Source Data files provided with this paper. The raw data can be made available upon request.

## References

[1] Slussarenko, S. & Pryde, G. J. Photonic quantum information processing: a concise review. *Appl. Phys. Rev.* **6**, 041303 10.1063/1.5115814 (2019).

[2] Monroe, C. et al. Programmable quantum simulations of spin systems with trapped ions. *Rev. Mod. Phys.* **93**, 025001 10.1103/RevModPhys.93.025001 (2021). 37540877

[3] Hilder, J. et al. Quantum internet in a nutshell – advancing quantum communication with ion traps. *arXiv* 10.48550/arXiv.2507.14383 (2025).

[4] Krantz, P. et al. A quantum engineer’s guide to superconducting qubits. *Appl. Phys. Rev.* **6**, 021318 10.1063/1.5089550 (2019).

[5] Kjaergaard, M. et al. Superconducting qubits: current state of play. *Annu. Rev. Condens. Matter Phys.* **11**, 369–395 10.1146/annurev-conmatphys-031119-050605 (2020).

[6] Jelezko, F. & Wrachtrup, J. Single defect centres in diamond: a review. *Phys. status solidi (a)* **203**, 3207–3225 10.1002/pssa.200671403 (2006).

[7] Loss, D. & DiVincenzo, D. P. Quantum computation with quantum dots. *Phys. Rev. A* **57**, 120–126 10.1103/PhysRevA.57.120 (1998).

[8] Gao, W. B., Imamoglu, A., Bernien, H. & Hanson, R. Coherent manipulation, measurement and entanglement of individual solid-state spins using optical fields. *Nat. Photonics* **9**, 363–373 10.1038/nphoton.2015.58 (2015).

[9] Togan, E. et al. Quantum entanglement between an optical photon and a solid-state spin qubit. *Nature* **466**, 730–734 10.1038/nature09256 (2010). 20686569

[10] Gao, W. B., Fallahi, P., Togan, E., Miguel-Sanchez, J. & Imamoglu, A. Observation of entanglement between a quantum dot spin and a single photon. *Nature* **491**, 426–430 10.1038/nature11573 (2012). 23151586

[11] de Greve, K. et al. Quantum-dot spin-photon entanglement via frequency downconversion to telecom wavelength. *Nature* **491**, 421–425 10.1038/nature11577 (2012). 23151585

[12] Lindner, N. H. & Rudolph, T. Proposal for pulsed on-demand sources of photonic cluster state strings. *Phys. Rev. Lett.* **103**, 113602 10.1103/PhysRevLett.103.113602 (2009). 19792371

[13] Thomas, P., Ruscio, L., Morin, O. & Rempe, G. Fusion of deterministically generated photonic graph states. *Nature* **629**, 567–572 10.1038/s41586-024-07357-5 (2024). PMC11096110 38720079

[14] Raussendorf, R., Browne, D. E. & Briegel, H. J. Measurement-based quantum computation on cluster states. *Phys. Rev. A* **68**, 022312 10.1103/PhysRevA.68.022312 (2003).

[15] Azuma, K., Tamaki, K. & Lo, H.-K. All-photonic quantum repeaters. *Nat. Commun.* **6**, 6787. 10.1038/ncomms7787 (2015). PMC4410623 25873153

[16] Buterakos, D., Barnes, E. & Economou, S. E. Deterministic generation of all-photonic quantum repeaters from solid-state emitters. *Phys. Rev. X* **7**, 041023 10.1103/PhysRevX.7.041023 (2017).

[17] Schwartz, I. et al. Deterministic generation of a cluster state of entangled photons. *Science* **354**, 434–437 (2016). 10.1126/science.aah4758 27608669

[18] Istrati, D. et al. Sequential generation of linear cluster states from a single photon emitter. *Nat. Commun.* **11**, 5501. 10.1038/s41467-020-19341-4 (2020). PMC7603328 33127924

[19] Li, J.-P. et al. Multiphoton graph states from a solid-state single-photon source. *ACS Photonics* **7**, 1603–1610 10.1021/acsphotonics.0c00192 (2020).

[20] Cogan, D., Su, Z.-E., Kenneth, O. & Gershoni, D. Deterministic generation of indistinguishable photons in a cluster state. *Nat. Photonics*10.1038/s41566-022-01152-2 (2023). PMC10091623 37064524

[21] Huet, H. et al. Deterministic and reconfigurable graph state generation with a single solid-state quantum emitter. *Nat. Commun.* **16**, 4337. 10.1038/s41467-025-59693-3 (2025). PMC12064760 40346067

[22] Thomas, P., Ruscio, L., Morin, O. & Rempe, G. Efficient generation of entangled multiphoton graph states from a single atom. *Nature* **608**, 677–681 10.1038/s41586-022-04987-5 (2022). PMC9402438 36002484

[23] Jackson, D. M. et al. Optimal purification of a spin ensemble by quantum-algorithmic feedback. *Phys. Rev. X* **12**, 031014 10.1103/PhysRevX.12.031014 (2022).

[24] Nguyen, G. N. et al. Enhanced electron-spin coherence in a GaAs quantum emitter. *Phys. Rev. Lett.* **131**, 210805 10.1103/PhysRevLett.131.210805 (2023). 38072607

[25] Zaporski, L. et al. Ideal refocusing of an optically active spin qubit under strong hyperfine interactions. *Nat. Nanotechnol.* **18**, 257–263 10.1038/s41565-022-01282-2 (2023). 36702953

[26] Appel, M. H. et al. A many-body quantum register for a spin qubit. *Nat. Phys.* **21**, 368–373 10.1038/s41567-024-02746-z (2025).

[27] Huthmacher, L. et al. Coherence of a dynamically decoupled quantum-dot hole spin. *Phys. Rev. B* **97**, 241413 10.1103/PhysRevB.97.241413 (2018).

[28] Pelc, J. S. et al. Downconversion quantum interface for a single quantum dot spin and 1550-nm single-photon channel. *Opt. Express* **20**, 27510–27519 10.1364/OE.20.027510 (2012). 23262701

[29] Zaske, S. et al. Visible-to-telecom quantum frequency conversion of light from a single quantum emitter. *Phys. Rev. Lett.* **109**, 147404 10.1103/PhysRevLett.109.147404 (2012). 23083285

[30] Kim, J. et al. Two-photon interference from an InAs quantum dot emitting in the telecom C-band. *Adv. Quantum Technol.* **8**, e2500069 10.1002/qute.202500069 (2025).

[31] Hauser, N. et al. Deterministic and highly indistinguishable single photons in the telecom C-band. *Nat. Commun.* **17**, 537. 10.1038/s41467-026-68336-0 (2026). PMC12804856 41530169

[32] Kaupp, J. et al. Purcell-enhanced single-photon emission in the telecom C-band. *Adv. Quantum Technol.* **6**, 2300242 10.1002/qute.202300242 (2023).

[33] Joos, R. et al. Coherently and incoherently pumped telecom C-band single-photon source with high brightness and indistinguishability. *Nano Lett.* **24**, 8626–8633 10.1021/acs.nanolett.4c01813 (2024). 38975638

[34] Holewa, P. et al. High-throughput quantum photonic devices emitting indistinguishable photons in the telecom C-band. *Nat. Commun.* **15**, 3358. 10.1038/s41467-024-47551-7 (2024). PMC11026509 38637520

[35] Dusanowski, L. et al. Optical charge injection and coherent control of a quantum-dot spin-qubit emitting at telecom wavelengths. *Nat. Commun.* **13**, 748. 10.1038/s41467-022-28328-2 (2022). PMC8826386 35136062

[36] Laccotripes, P. et al. Spin-photon entanglement with direct photon emission in the telecom C-band. *Nat. Commun.* **15**, 9740. 10.1038/s41467-024-53964-1 (2024). PMC11554671 39528490

[37] Laccotripes, P. et al. An entangled photon source for the telecom C-band based on a semiconductor-confined spin. *arXiv* 10.48550/arXiv.2507.01648 (2025).

[38] Peniakov, G. et al. Initialization of neutral and charged exciton spin states in a telecom-emitting quantum dot. *Phys. Rev. B* **112**, 085422 10.1103/3qh7-b696 (2025).

[39] Wasiluk, M. et al. Probing electron spin dynamics in single telecom InAs(P)/InP quantum dots using the Hanle effect.* Appl. Phys. Lett.* **127**, 204001 10.1063/5.0291044 (2025).

[40] Huang, J. et al. Optical spin tomography in a telecom C-band quantum dot. *Optica Quantum*** 4**, 10.1364/OPTICAQ.589319 (2026).

[41] Behrends, R. et al. Highly indistinguishable single-photons at 1550 nm from a two-photon resonantly excited Purcell-enhanced quantum dot. *arXiv* 10.48550/arXiv.2602.06140 (2026).

[42] Peniakov, G. et al. Polarized and unpolarized emission from a single emitter in a bullseye resonator. *Laser Photonics Rev.* **18**, 2300835 10.1002/lpor.202300835 (2024).

[43] Buchinger, Q. et al. Deterministic quantum dot cavity placement using hyperspectral imaging with high spatial accuracy and precision. *Nano Converg.* **12**, 36. 10.1186/s40580-025-00501-5 (2025). PMC12267725 40668472

[44] Gong, M. et al. Electronic structure of self-assembled InAs/InP quantum dots: Comparison with self-assembled InAs/GaAs quantum dots. *Phys. Rev. B* **77**, 045326 10.1103/PhysRevB.77.045326 (2008).

[45] Holewa, P. et al. Optical and electronic properties of low-density InAs/InP quantum-dot-like structures designed for single-photon emitters at telecom wavelengths. *Phys. Rev. B* **101**, 195304 10.1103/PhysRevB.101.195304 (2020).

[46] Coste, N. et al. Probing the dynamics and coherence of a semiconductor hole spin via acoustic phonon-assisted excitation. *Quantum Sci. Technol.* **8**, 025021 10.1088/2058-9565/acbd6a (2023).

[47] Serov, Y. et al. Hidden anisotropy controls spin-photon entanglement in a charged quantum dot. *Phys. Rev. Appl.* **23**, 044019 (2025).

[48] Merkulov, I. A., Efros, A.lL. & Rosen, M. Electron spin relaxation by nuclei in semiconductor quantum dots. *Phys. Rev. B* **65**, 205309 10.1103/PhysRevB.65.205309 (2002).

[49] Testelin, C., Bernardot, F., Eble, B. & Chamarro, M. Hole–spin dephasing time associated with hyperfine interaction in quantum dots. *Phys. Rev. B* **79**, 195440 10.1103/PhysRevB.79.195440 (2009).

[50] Houel, J. et al. High resolution coherent population trapping on a single hole spin in a semiconductor quantum dot. *Phys. Rev. Lett.* **112**, 107401 10.1103/PhysRevLett.112.107401 (2014). 24679326

[51] Belykh, V. V. et al. Electron and hole g factors in InAs/InAlGaAs self-assembled quantum dots emitting at telecom wavelengths. *Phys. Rev. B* **92**, 165307 10.1103/PhysRevB.92.165307 (2015).

[52] Belykh, V. V. et al. Large anisotropies of electron and hole g factors in infrared-emitting InAs/InAlGaAs self-assembled quantum dots. *Phys. Rev. B* **93**, 125302 10.1103/PhysRevB.93.125302 (2016).

[53] Bechtold, A. et al. Three-stage decoherence dynamics of an electron spin qubit in an optically active quantum dot. *Nat. Phys.* **11**, 1005–1008 10.1038/nphys3470 (2015).

[54] Cogan, D. et al. Depolarization of electronic spin qubits confined in semiconductor quantum dots. *Phys. Rev. X* **8**, 041050 10.1103/PhysRevX.8.041050 (2018).

[55] Eble, B. et al. Hole–nuclear spin interaction in quantum dots. *Phys. Rev. Lett.* **102**, 146601 10.1103/PhysRevLett.102.146601 (2009). 19392463

[56] Evers, E. et al. Hole spin coherence in InAs/InAlGaAs self-assembled quantum dots emitting at telecom wavelengths. *Phys. status solidi (b)* **262**, 2400174 10.1002/pssb.202400174 (2025).

