# Supplementary material for: A spin-photon interface in the telecom C-band with long hole spin dephasing time
Source: Nat Commun. 2026 Sep 2;17:10458. doi: 10.1038/s41467-026-77282-w (PMC13635138; doi:10.1038/s41467-026-77282-w)
Supplement: Supplementary file 1 [file 41467_2026_77282_MOESM1_ESM.pdf]

Supplementary Material

A Spin-Photon Interface in the Telecom C-Band with Long Hole Spin Dephasing Time

Johannes M. Michl[1,*,†], Reza Hekmati[1,*,†], Mohamed Helal[1], Giora Peniakov[1], Yorick Reum[1], Jochen Kaupp[1], Quirin Buchinger[1], Jaewon Kim[1,2], Andreas T. Pfenning[1], Yong-Hoon Cho[2], Sven Höfling[1], Tobias Huber-Loyola[1,3]

[1] Julius-Maximilians-Universität Würzburg, Physikalisches Institut and Würzburg-Dresden Cluster of Excellence ctd.qmat, Lehrstuhl für Technische Physik, Am Hubland, 97074 Würzburg, Deutschland.

[2] Department of Physics, Korea Advanced Institute of Science and Technology (KAIST), 34141 Daejeon, Republic of Korea.

[3] Karlsruhe Institute of Technology, Institute of Photonics and Quantum Electronics (IPQ) and Center for Integrated Quantum Science and Technology (IQST), Germany.

[*] Corresponding authors: johannes.michl@uni-wuerzburg.de; reza.hekmati@uni-wuerzburg.de

[†] These authors contributed equally to this work.

Contents

S1 Details on basic characterization measurements and setup **2**

S1.1 Device and spectrum 2

S1.2 Identification of exciton and trion 3

S1.3 PLE data 4

S1.4 Setup sketch 5

S2 Additional datasets on polarization resolved lifetime measurements **6**

S2.1 Polarization correction with waveplates 6

S2.2 Excited state spin precession with alternating excitation polarization 7

S2.3 Comparison of polarization memory achieved by p-shell and LA-phonon excitation 8

S3 Extended datasets on two-photon correlation experiments with cw-excitation **9**

S3.1 Magnetic field dependent spin dephasing time 9

S3.2 Comparison of linear excitation vs. circular excitation polarization 12

S3.3 Power dependent spin dephasing time 13

S3.4 Discussion of extracted parameter alpha 14

S4 Additional information on analysis of pulsed T_2^* measurement **15**

S5 Magnetic field configurations and selection rules **17**

References **19**

S1 Details on basic characterization measurements and setup

S1.1 Device and spectrum

Fig. S1 shows the spectrum of the QD studied in this work under above-bandgap excitation for small excitation powers (black, single lines) as well as for high excitation powers (red). The latter spectrum shows the resonance of the CBG cavity.

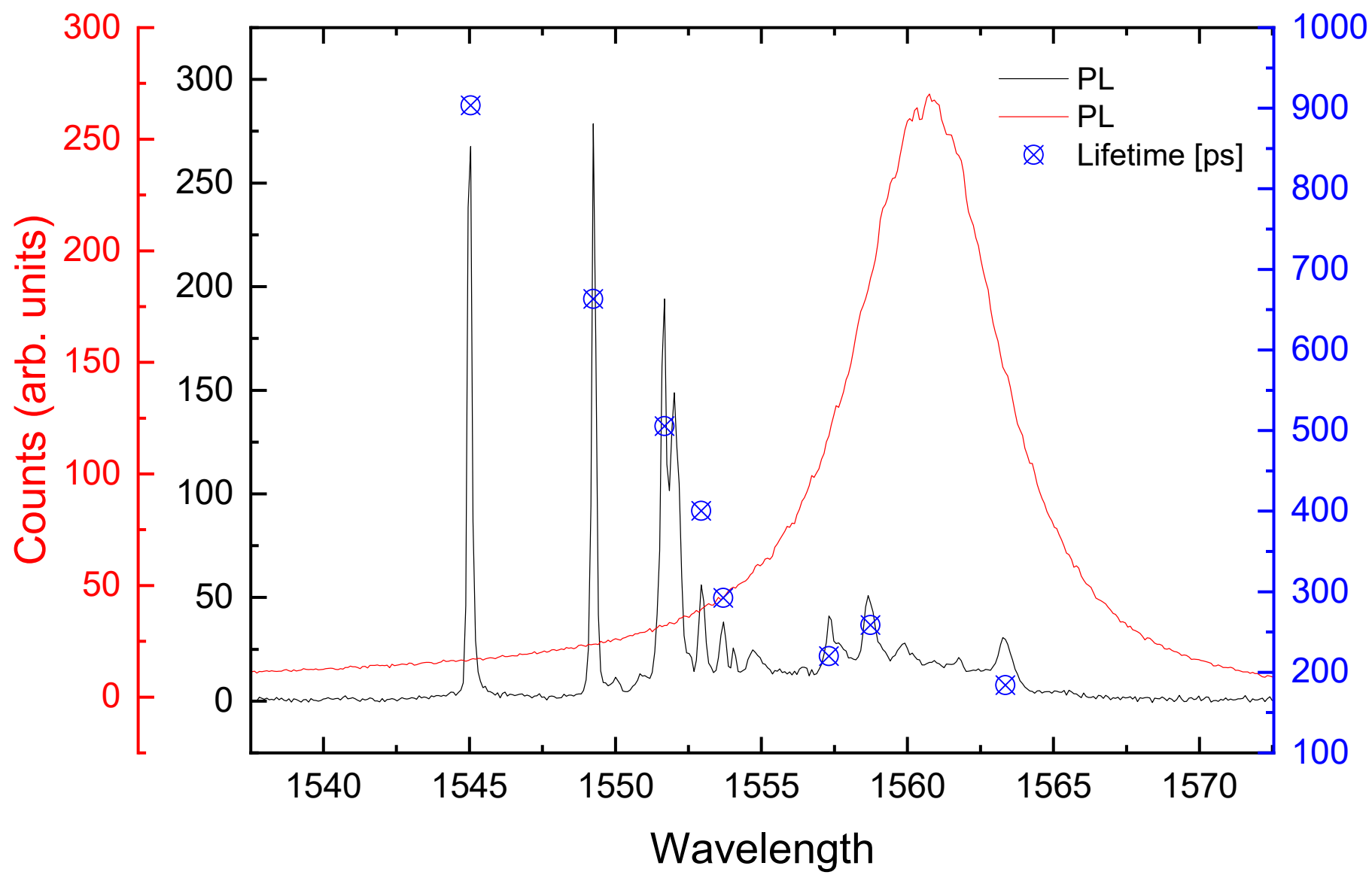

Figure S1: μ-PL spectrum and cavity enhanced radiative lifetime measurements. Black: Spectrum under above-bandgap laser illumination. Two prominent emission lines appear below 1550 nm. Red: Cavity emission under strong laser pumping. The cavity is red-shifted around 10 nm from the targeted center wavelength. Blue: Measurements of the radiative lifetime at the position of prominent PL-features. The lifetime significantly decreases for lines that couple to the cavity. Green: Purcell factor calculated from the decreased radiative decay times. The Purcell enhancement follows the cavity's spectral shape.

S1.2 Identification of exciton and trion

The positive trion studied in this work is located at $\sim 1549\,\text{nm}$ (cf. Fig. S2 (a)). We could identify the exciton and trion in our sample by means of power- and polarization series using above-bandgap continuous-wave (cw) excitation. One hallmark of a trion is the lack of any observable fine-structure splitting (FSS) which can become apparent when rotating the polarization angle in the detection. While the neighboring exciton spectral line showed a clear FSS of $\sim 71\,\mu\text{eV}$, the trion line investigated here showed no splitting as is depicted in Fig. S2 (b-d). For both, X^0 and X^+, we furthermore measured a power-law coefficient of $\alpha \sim 1.2$ according to $I(P) \propto P^{\alpha}$.

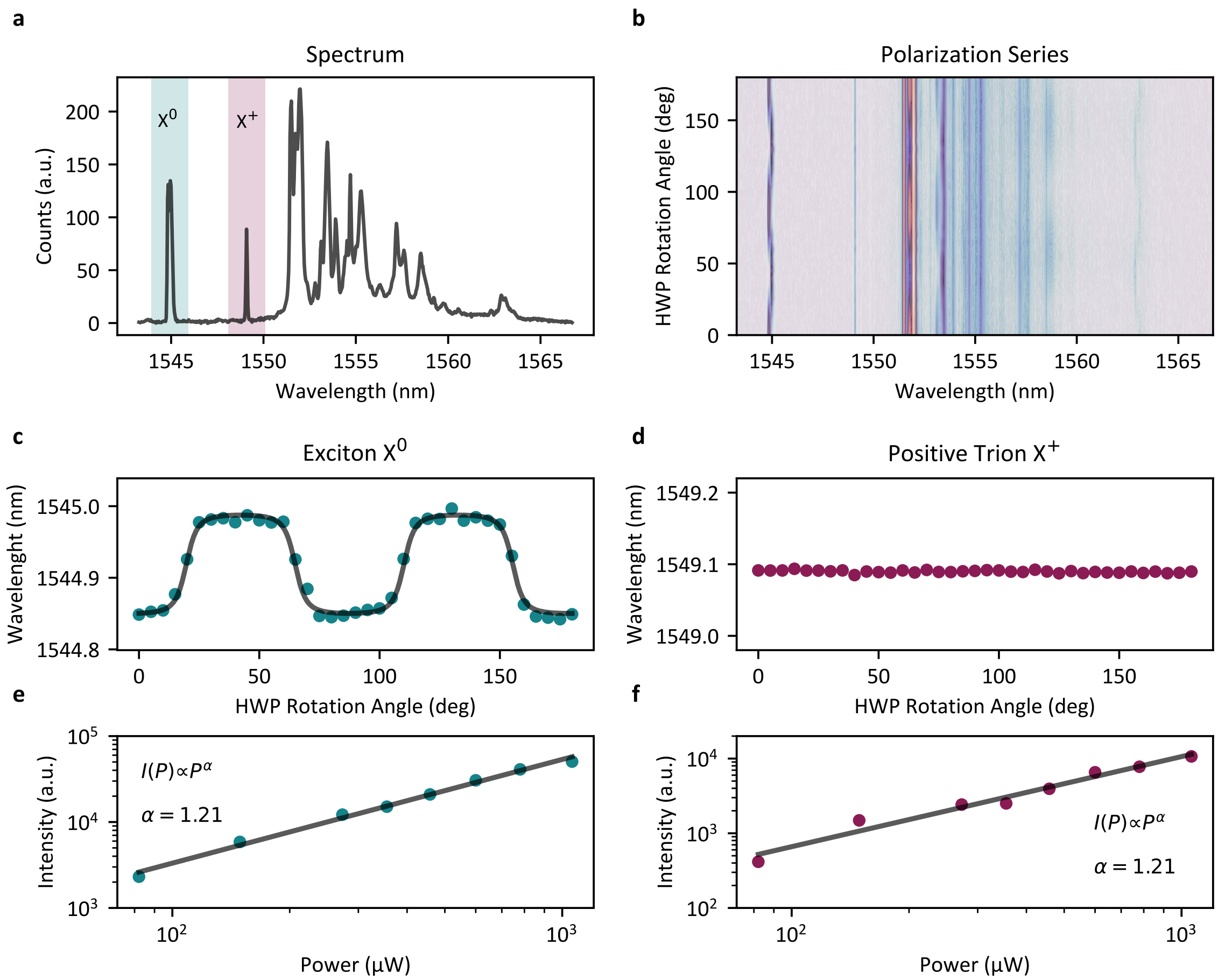

Figure S2: Analysis of peak position and intensity under varying excitation powers and detection polarizations. (a) QD spectrum with the exciton and trion line highlighted. (b) Colormap constructed from all spectra of a polarization series, with the HWP angle in the detection tuned from (0–180)°. (c-d) Peak position of X^0 and X^+ from polarization series. The exciton shows a fine-structure-splitting, whereas the trion wavelength remains polarization-independent. (e-f) Respective power dependent peak intensities. Both fitted lines showcase a power-law coefficient of ~ 1.2.

S1.3 PLE data

The exciting laser was scanned from 1465 nm to 1540 nm and a spectrum of the QD was acquired for each excitation wavelength, producing the colormap on the left side of Fig. S3. A vertical cut at the trion wavelength $\lambda = 1549.08$ nm reveals a rich spectrum of resonances for the trion (blue plot at the upper right) with the most notably bright resonance at $\lambda_{exc} = 1520.76$ nm.

Exciting with this laser energy yields the PL that is depicted in the lower right figure and which can be illustrated by a horizontal cut through the colormap (indicated by green stripe). The trion transition stands out against the background, with the exception of a weak emission at the exciton energy (∼1545 nm). In most of the measurements presented in this work, this excitation wavelength is used and the trion is spectrally filtered to exclude any PL collection from e.g. the exciton.

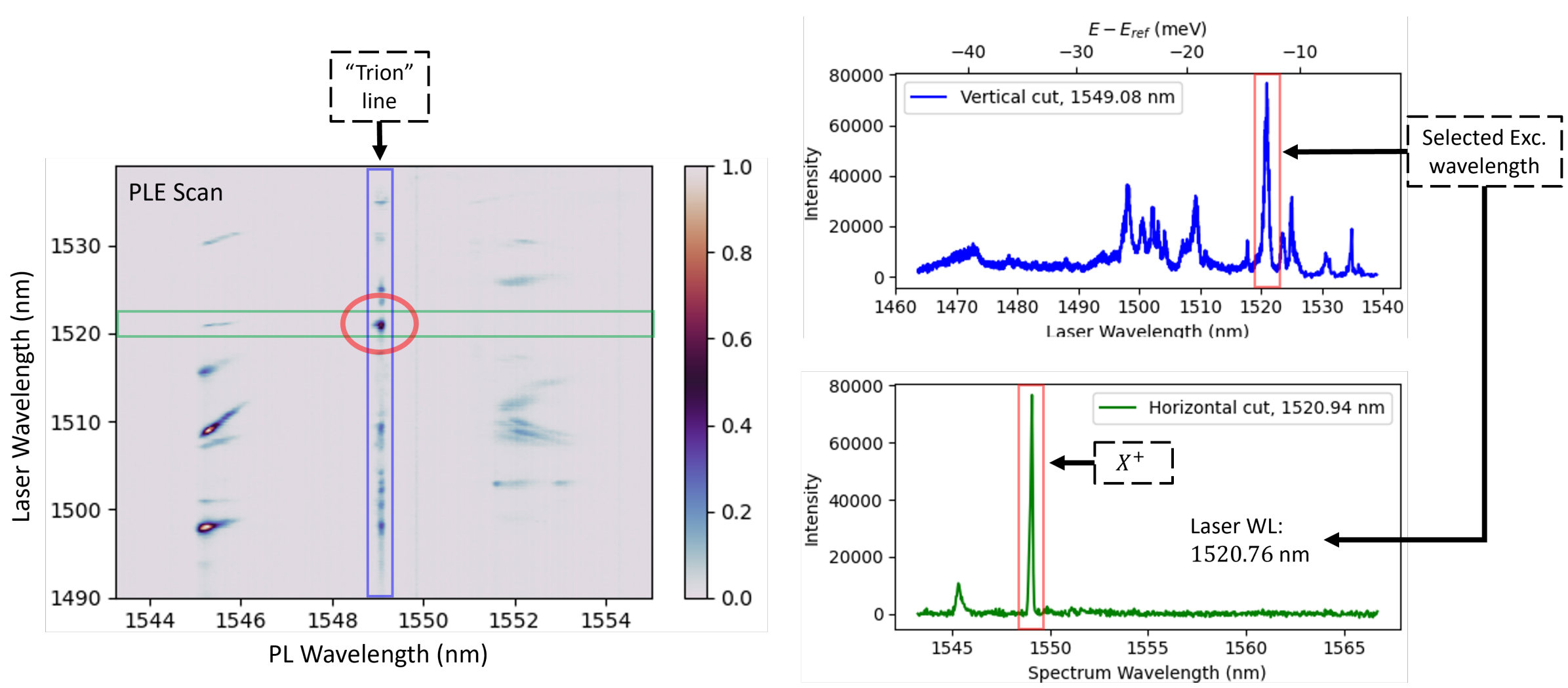

Figure S3: PLE scan. Left side: Colormap showcasing the resonances of the QD spectral emission lines for the scanned excitation laser wavelengths. Right side: Vertical (blue) and horizontal (green) cuts through a resonance found at $\lambda_{exc} \approx 1521$ nm.

S1.4 Setup sketch

This section provides a sketch of the optical setup that was used for the results presented in this work. Please refer to the methods section of the main text for detailed information about how the setup was used.

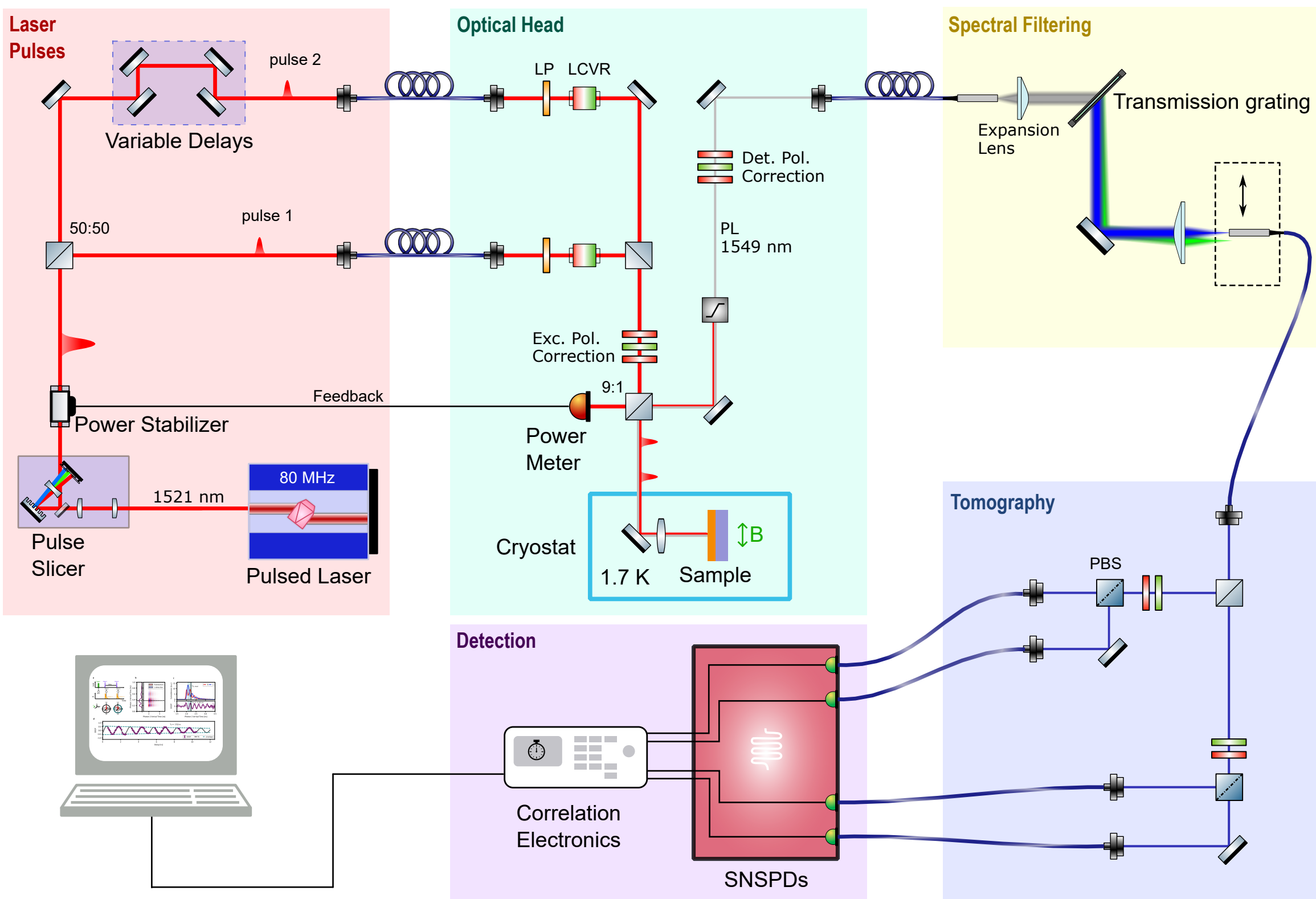

Figure S4: Sketch of the optical setup. The main building blocks of the setup are: (i) module for laser pulses (red). (ii) Optical head, including cryostat with magnet. (iii) Module for spectral filtering, e.g. through transmission grating monochromator or other fiber coupled bandpass filters. (iv) Tomography module. (v) Detection module.

S2 Additional datasets on polarization resolved lifetime measurements

S2.1 Polarization correction with waveplates

Details on the polarization correction and calibration using waveplates is provided in the methods section. Fig. S5 displays the results of one calibration which was performed iteratively until oscillations in the time trace could only be observed when exciting and detecting in the R/L polarization basis.

For the polarization corrections, the excitation- and detection polarization correction modules were used which are illustrated in in the setup sketch Fig. S4 from the previous section.

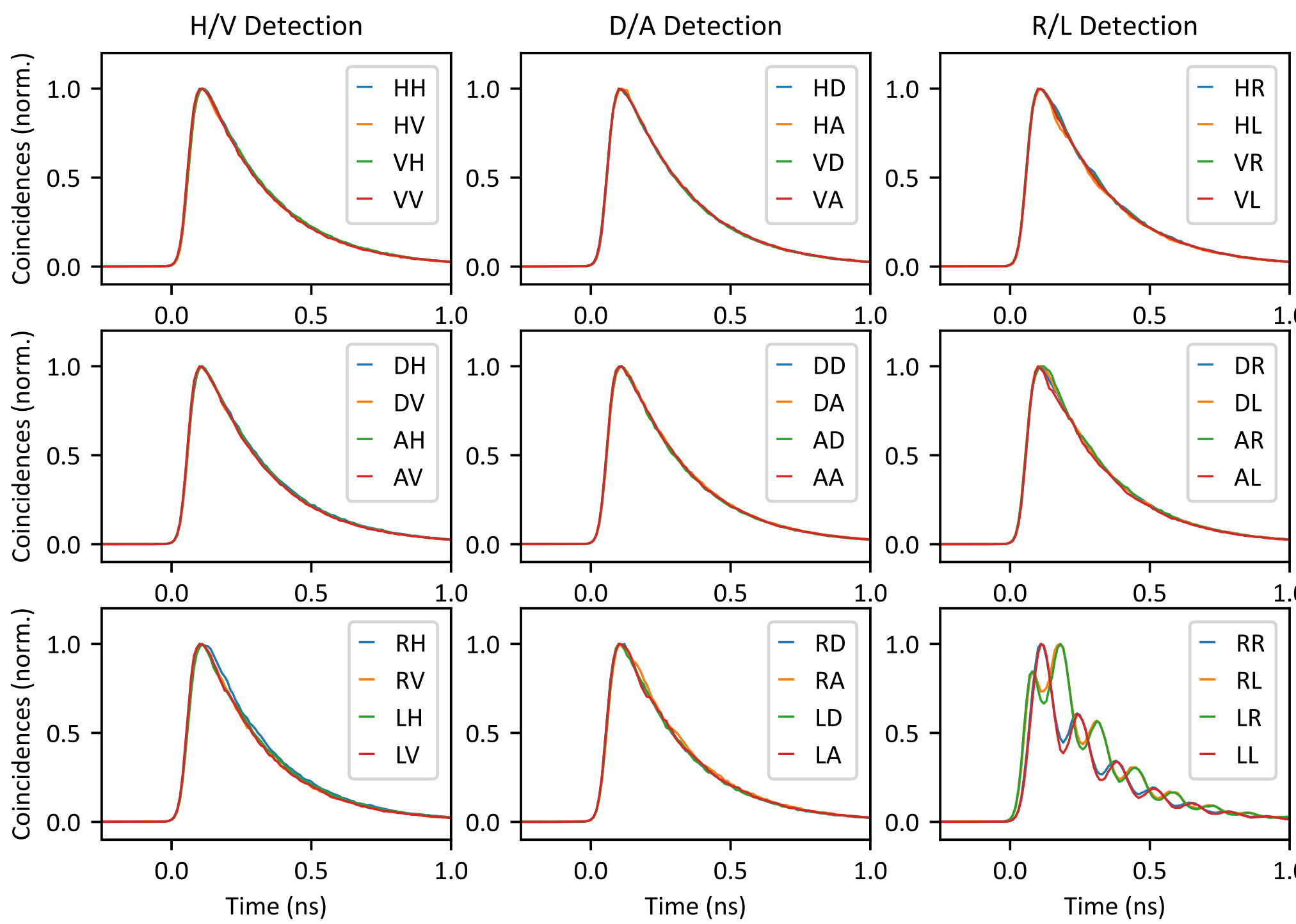

Figure S5: Optimization on lifetime oscillations. The labeling of each subset of data follows the convention *excitation-polarization/detection-polarization*, so RL e.g. means a excitation with R polarized laser and a detection of L polarized PL.

S2.2 Excited state spin precession with alternating excitation polarization

Excitation of quantum dots with a constant circular polarization can lead to dynamic nuclear polarization (DNP) which can create effects that skew the observed oscillations that are used in this work to directly monitor the evolution of excited- and ground-level spin states in a transverse magnetic field.

To exclude that trion level spin oscillations that we observe when measuring its polarization resolved time trace (cf. main text figure Fig. 1 (d)) are affected by DNP, we have conducted a control experiment in which we utilize the capability of our optical setup to split the laser pulse from one repetition cycle into two excitation paths with individual polarization control. In Fig. S6 we directly compare two cases: i) exciting only with R-polarization in both pulses (panel (a)) and ii) switching between R- and L-polarized excitation with each pulse, by changing the polarization of the second excitation path to L (panel (b)). In the bottom panels (c) and (d) we plot the DOCP as calculated from the respective top panels.

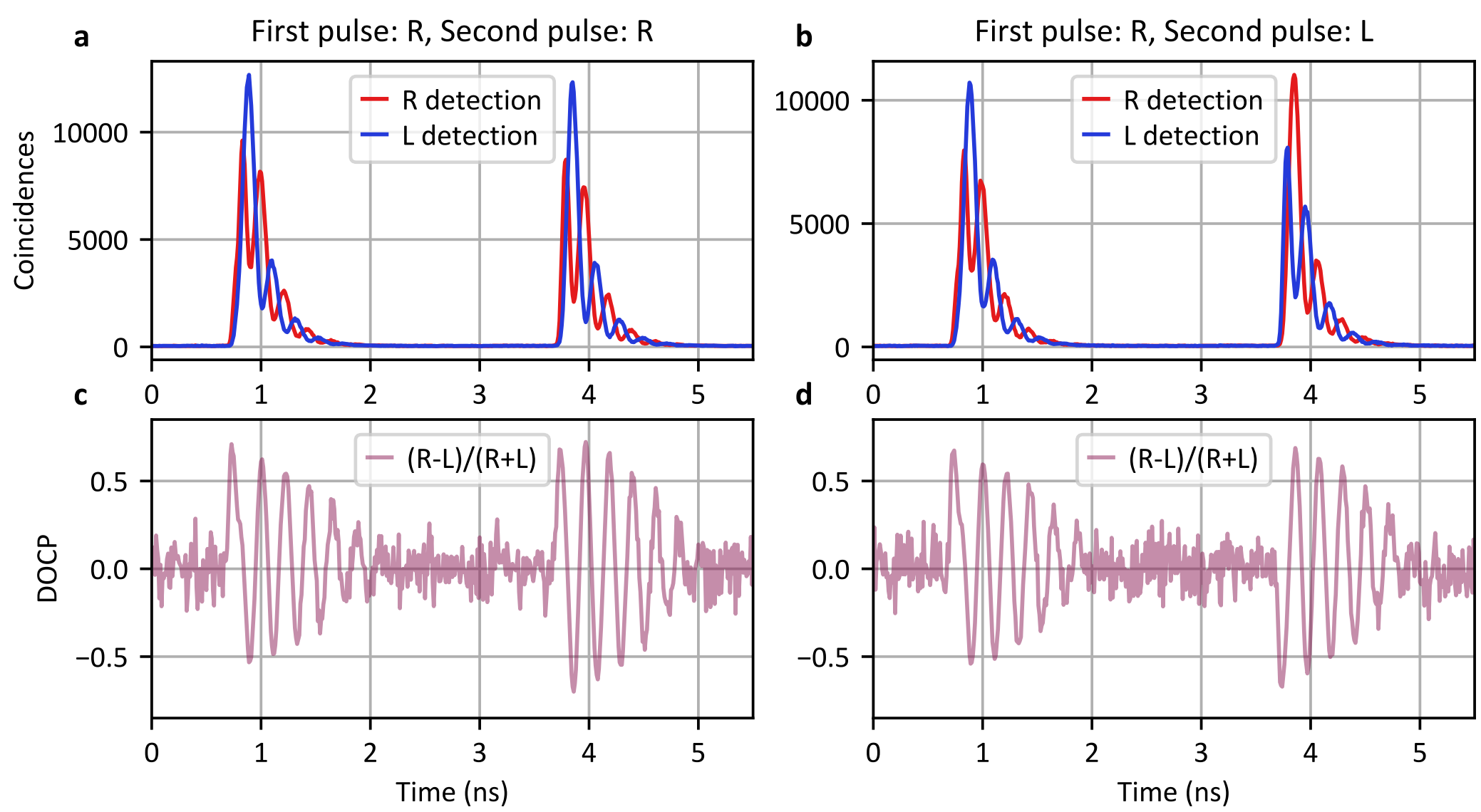

Figure S6: Polarization-resolved time trace for co- and cross-polarized excitation pulses. To exclude the impact of dynamic nuclear polarization through constant excitation with R- or L- circularly polarized laser, we compare the oscillating time traces for two cases. **(a)** Excitation with two R-polarized laser pulses. **(b)** Alternating excitation with an R-polarized laser pulse, followed by an L-polarized laser pulse. **(c, d)** Degree of polarization, calculated from (a) and (b) respectively. It can be easily observed that switching the polarization of the second laser flips the phase of the second oscillation signal by $\pi/2$. No difference in the dephasing time constant of the oscillations can be observed.

Comparing (c) and (d), it can be easily observed that the DOCP is mostly preserved during the radiative decay for both cases. The most significant difference is that the DOCP oscillation in (d) resulting from the second laser pulse starts with a π offset for compared to its counterpart in (c). This phase flip directly illustrates the opposite spin initialization (into $|T_{\uparrow}\rangle$ instead of $|T_{\downarrow}\rangle$).

S2.3 Comparison of polarization memory achieved by p-shell and LA-phonon excitation

Throughout this work we utilize quasi-resonant p-shell excitation due to its simple experimental realization and robust spin selective excitation. Next to p-shell there are other known spin-preserving excitation schemes like LA-phonon excitation, which is commonly used for similar spin studies. We have conducted an experiment using LA-phonon excitation to compare its spin initialization fidelity with p-shell excitation. Fig. S7 shows two polarization resolved time trace measurements comparing both approaches. We calculated the maximal achievable DOCP for both datasets and could observe an improvement of the polarization memory from 0.92 (p-shell) to 0.97 for LA-phonon excitation.

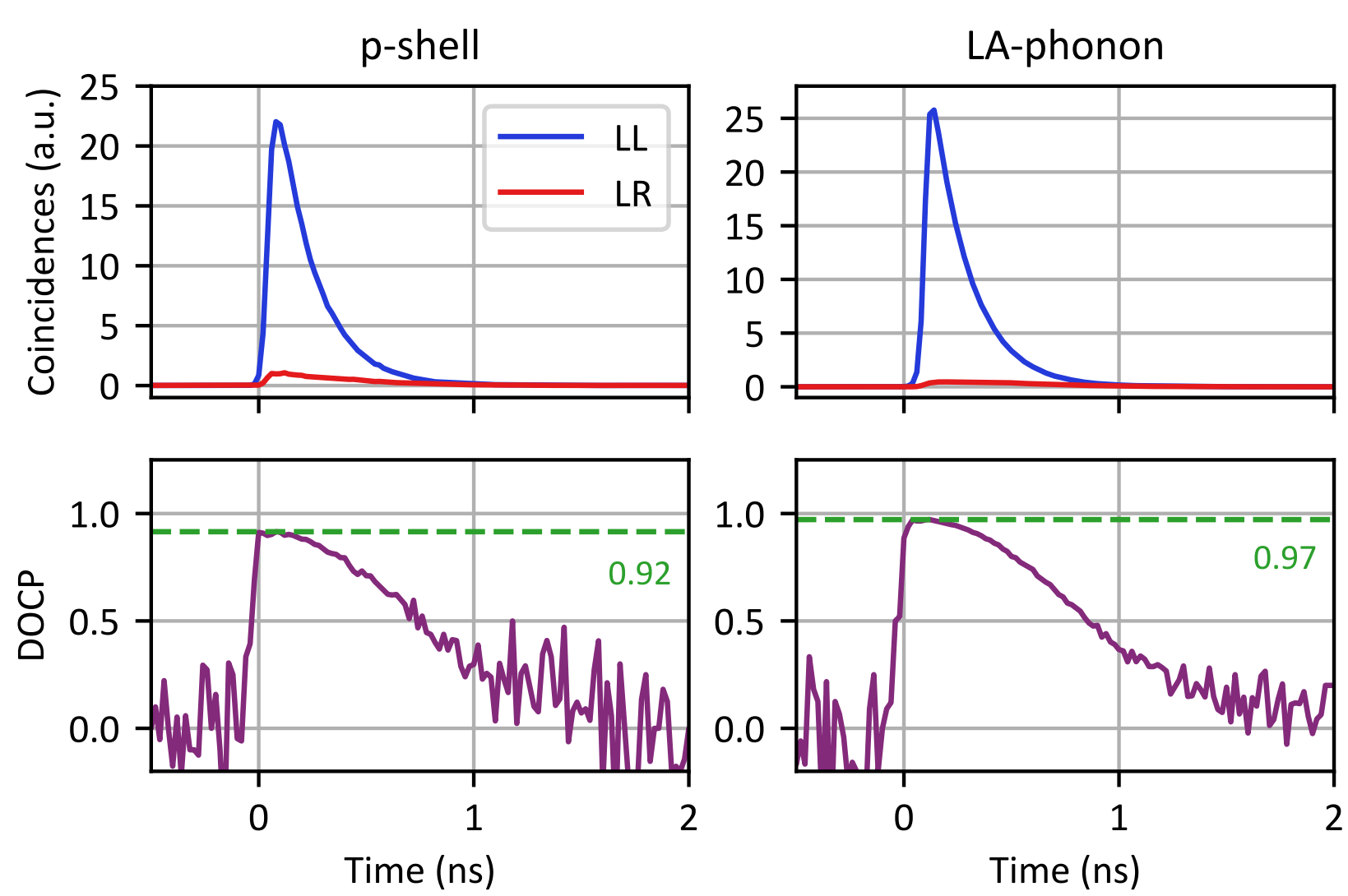

Figure S7: Comparison of polarization memory achieved by p-shell and LA-phonon excitation. (Left, top) PL time trace under co-polarized (LL) and cross-polarized (LR) excitation/detection for p-shell excitation. (Right, top) Same measurement for LA-phonon assisted excitation. (Bottom) Degree of polarization calculated from the respective top panels, with the maximal reached value of the DOCP marked by a dashed, green line. The maximal DOCP is 0.92 for p-shell and 0.97 for LA-phonon.

S3 Extended datasets on two-photon correlation experiments with cw-excitation

Here, we provide additional information and extended datasets for the polarization resolved autocorrelation measurement under continuous-wave excitation, that form the content of Fig. 2 of the main text. While the main text figure focuses on conveying the essential information, this sections explains in more detail how the data analysis and fits were performed and includes measurements using circularly polarized excitation.

For each data point we fitted the degree of polarization DOCP that was calculated from two datasets in which the polarization was switched from parallel to orthogonal polarizations. The damped oscillations of the degree of polarization were fitted using the following function:

$$DOCP(t) = C + A\ e^{-(\frac{|t-t_0|}{\tau})^{\alpha}} \cos\left(2\pi f t + \varphi\right). \tag{S1}$$

Using the absolute value $|t - t_0|$ ensures a fit that is symmetric around $t = 0$. The stretching parameter α was left free to account empirically for deviations from a purely Gaussian Overhauser-field-induced decay, since multiple dephasing channels may contribute to the damping of the hole-spin precession. Additionally, frequency filtering was applied via Fourier transformation to clean up the signal from the ground state spin precession for better fitting. Exemplary dataset featuring data both in the time as well as in the frequency domain are shown in the following subsection.

S3.1 Magnetic field dependent spin dephasing time

Exciting with H-polarized laser, the degree of polarization was calculated via $DOCP = (RR - RL)/(RR + RL)$, with RL e.g. being detection with R in channel 1 and L in channel 2. The in-plane magnetic field was varied from 5 mT to 90 mT and for each field the DOCP was fitted using Eq. S1, after filtering the Fourier transformed DOCP in the frequency domain.

Fig. S8 shows the fitting for four magnetic fields (5 mT, 10 mT, 15 mT and 20 mT). The right column shows the frequency domain for the above fields. There is a low frequency component

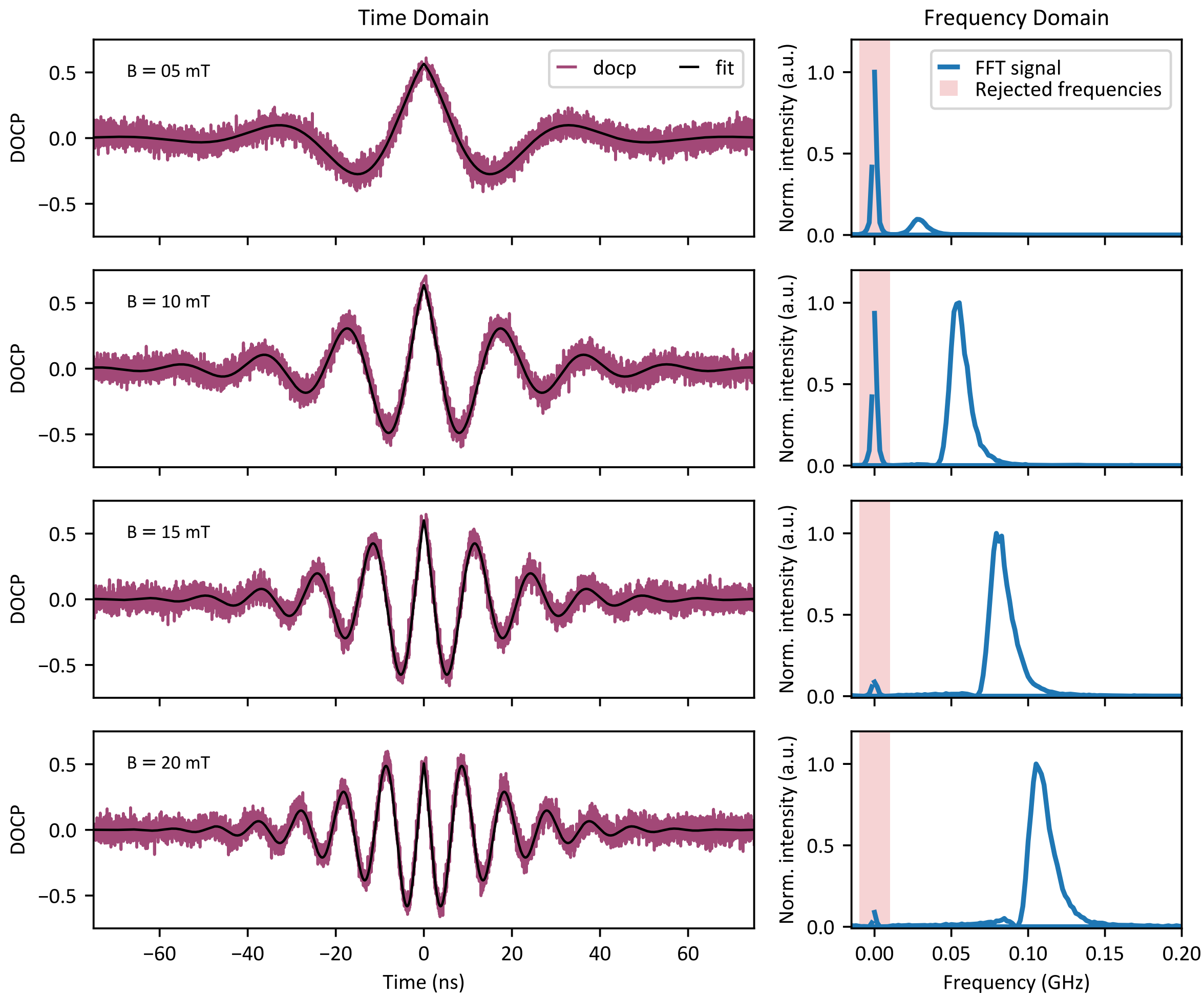

Figure S8: Magnetic field series of cw-g2. Left column: Fits of the oscillating DOCP, depending on magnetic field. From the fits, the dephasing time τ, parameter α and oscillation frequency f are extracted. Shown are four fitted datasets ranging from (5–20) mT. The fitting was performed the same way for the datasets from (50–90) mT. Right column: Fourier transformation of the signals from the left column, showcasing the shift of the Fourier signal frequency with magnetic field. For small magnetic fields, a low frequency component with $|f| \leq 10\,\mathrm{MHz}$ emerges. Filtering in the frequency domain with subsequent inverse Fourier transformation is applied in order to reject this signal.

at $|f| \leq 10\,\mathrm{MHz}$ emerging that increasing in relative intensity compared to the spin precession signal and even dominates for $B = 5\,\mathrm{mT}$. To isolate the spin precession, these frequencies are suppressed as indicated by the red background in the plots and only frequency components with $|f| > 10\,\mathrm{MHz}$ are transformed back to the time domain. The resulting filtered DOCP, including fits, is displayed in the left column. From these fits, the dephasing times τ and frequencies f are extracted, that are plotted in the main text figure Fig. 2 (d).

Fig. S9 displays a zoom-in on the central fit region for two datasets $B = 5\,\mathrm{mT}$ and $B = 80\,\mathrm{mT}$.

The unprocessed data, after calculating the DOCP from two raw datasets, are plotted in the left column. The middle column shows the same data after frequency filtering. It is clearly visible for the 5 mT data that this step removes a bias of the DOCP towards positive values that would otherwise have artificially reduced the fitted dephasing time drastically. The right column shows the next step in the analysis before fitting that we applied, which is to exclude data points in a window of ±460 ps around t_0 for the fit. The 80 mT data prominently show the antibunching dip which occurs naturally in the signal because of the low multi-photon probability of the measured photons. To avoid distortion of the fit, we exclude this dip by omitting a window of twice the X^+ radiative lifetime.

Note that the fitted spin precession does not start with a phase of $\varphi = 0$ at times $t = 0$. We have observed that this is due to a phase-addition during re-excitation, that occurs for linearly polarized excitation. We could observe that this phase can be tuned between $0 \leq \varphi \leq \pi$ when changing between linear polarizations H, V, D, A.

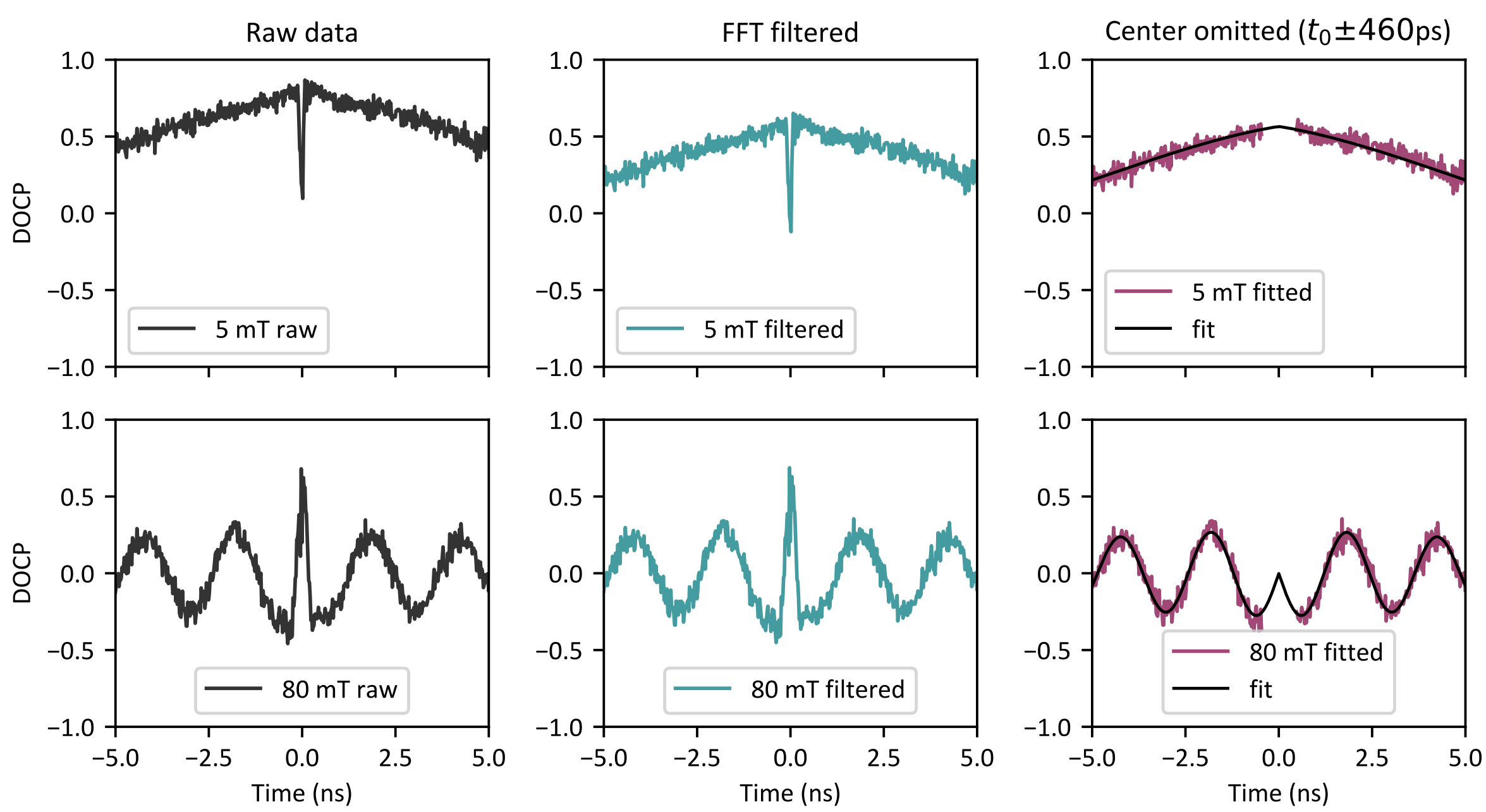

Figure S9: Zoom in to the central fit region. Shown are the main stages of data processing for two datasets, 5 mT (first row) and 80 mT (second row). Left column: DOCP as calculated from the raw data following $DOCP = (R - L)/(R + L)$. The strong low frequency component at 5 mT creates a visible bias. Middle column: Signal with applied frequency filtering, rejecting all frequencies $f < 10$ MHz. Right column: Fit fitting, a window of ±460 ps is omitted from the data, since the antibunching dip in the correlation data around t_0 hinders proper fit convergence.

S3.2 Comparison of linear excitation vs. circular excitation polarization

We have conducted the experiments presented in Fig. 2 of the main text using both linear excitation with a H-polarized laser as well as using circular excitations with R and L. Fig. S10 compares the dephasing times and the initial degree of polarization at $t = 0$ that result from both excitation methods. Panel Fig. S10 (a) displays the dephasing times τ. It clearly shows a decreasing of the spin coherence with increasing magnetic field, with a plateau that is reached for magnetic fields $B \leq 20\,\mathrm{mT}$ for H-polarization. Overall, the values of τ are slightly higher using linearly polarized excitation.

The right hand side panel (b) on the other hand displays the initial degree of polarization $DOCP_0$ for each magnetic field that marks the starting point of the damped oscillations at $t = 0$. Here, we can observe that higher initial DOCP amplitudes are maintained using circular polarization. Additionally, the $DOCP_0$ for magnetic fields > 10mT decays faster for H-polarized excitation as compared to $L(R)$-polarized excitation. Whether this effect arises solely from spin-selective initialization governed by the trion polarization selection rules or instead reflects dynamic nuclear polarization induced by continuous circularly polarized excitation remains inconclusive at this point.

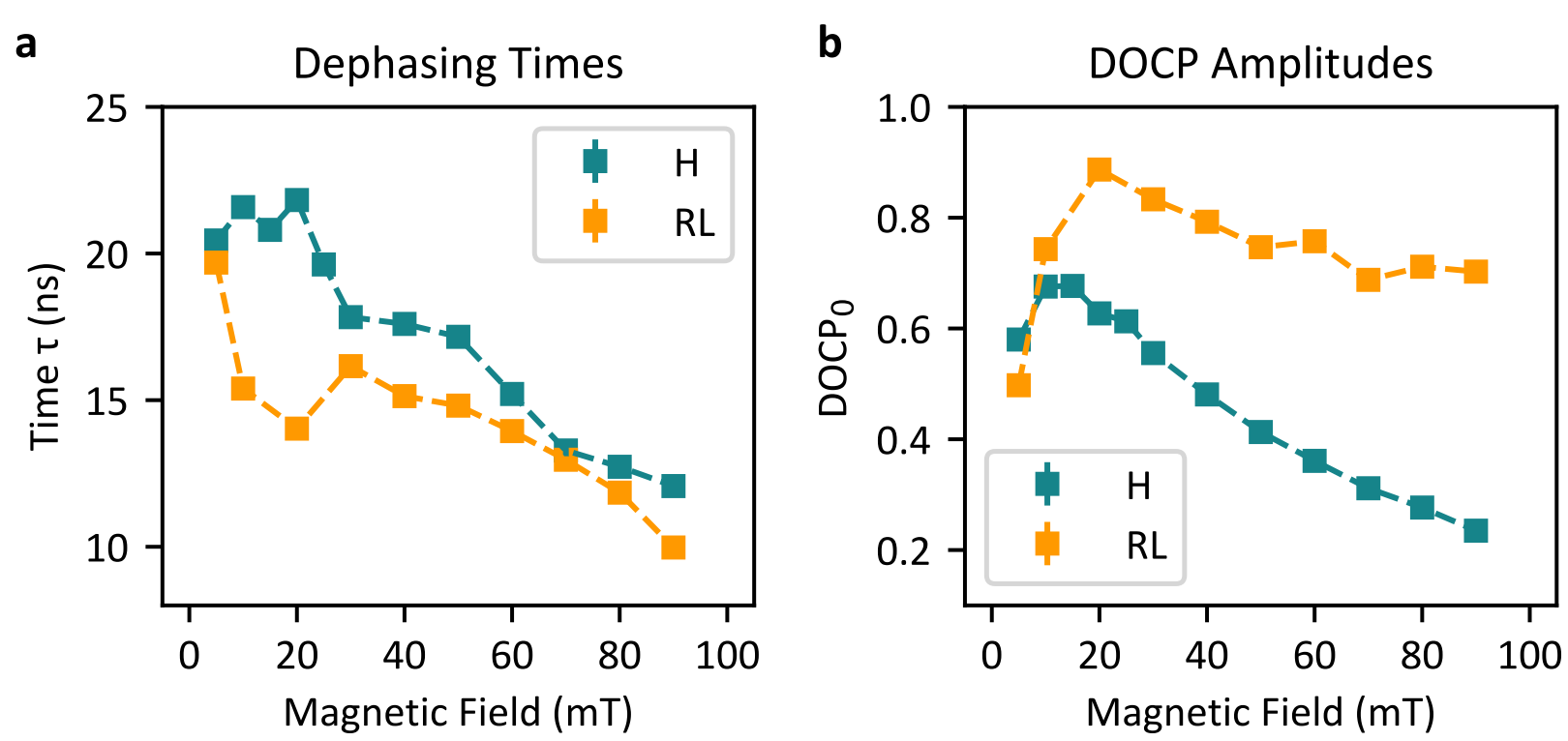

Figure S10: Magnetic field dependent dephasing times for linearly polarized and circularly polarized excitation. The left panel **(a)** depicts the magnetic field dependent dephasing times for H-polarized excitation as well as for circularly polarized laser illumination. The extracted time constant τ decreases with increasing B-field, for both polarizations. **(b)** shows the fitted initial degree of polarization at $t = 0$, before dephasing. Using circular excitation, the DOCP remains higher values whereas it drops faster with increasing field for the H-polarized excitation.

S3.3 Power dependent spin dephasing time

In this supplement section, we show fits of the spin precession under continuous-wave excitation for varying excitation powers, using Eq. S1. We follow the analysis described for the magnetic field series above (supplement section S3.1) that includes Fourier filtering and omitting a window around t_0 for the fit.

Here, too, we performed the experiments using both linear as well as circular polarization in the excitation. Fig. S11 depicts four fits using linearly polarized excitation with H. We limit this figure to display the fits for $P = 1\,\mu W$, $2.0\,\mu W$, $5.0\,\mu W$ and $10.0\,\mu W$. The extracted dephasing times from the full set of data, which includes powers ranging from $(1–20)\,\mu W$, forms the sub-panel (d) of main text figure Fig. 2. Increasing the excitation power from $1\,\mu W$ to $2\,\mu W$ drastically reduces the fitted dephasing time of the observable oscillations (cf. Fig. S11).

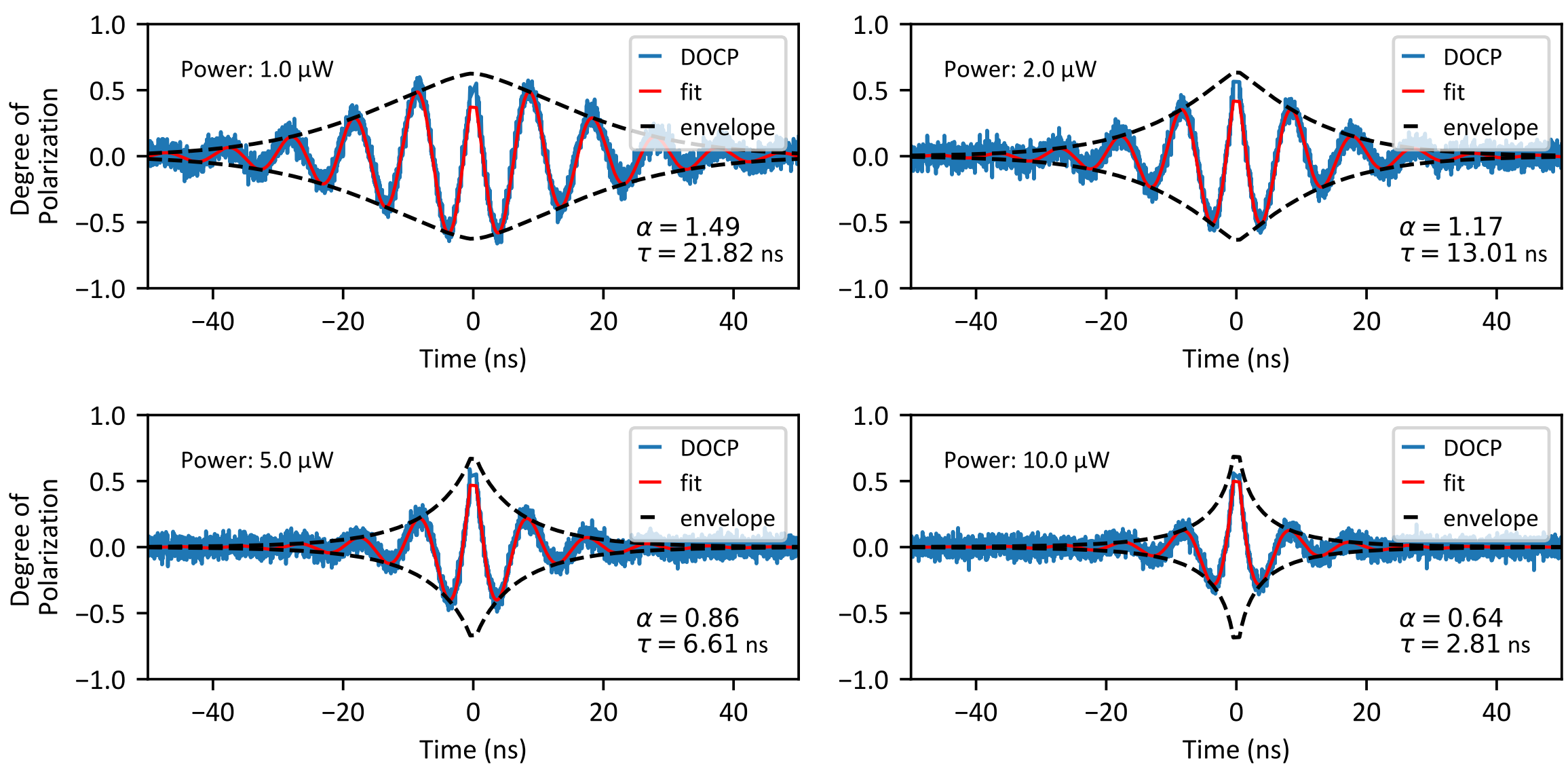

Figure S11: Power series of cw-g2 using H-polarized excitation. The power is increased from the upper left column to the lower right column. For each power, RR and RL datasets were acquired and the DOCP was calculated and fitted, extracting the dephasing time from the exponentially decaying envelope.

Exciting with circular polarizations, four sets of data are analyzed, with $P = 0.5\,\mu W$, $2.0\,\mu W$, $5.0\,\mu W$, $10.0\,\mu W$. The fits are displayed in Fig. S12

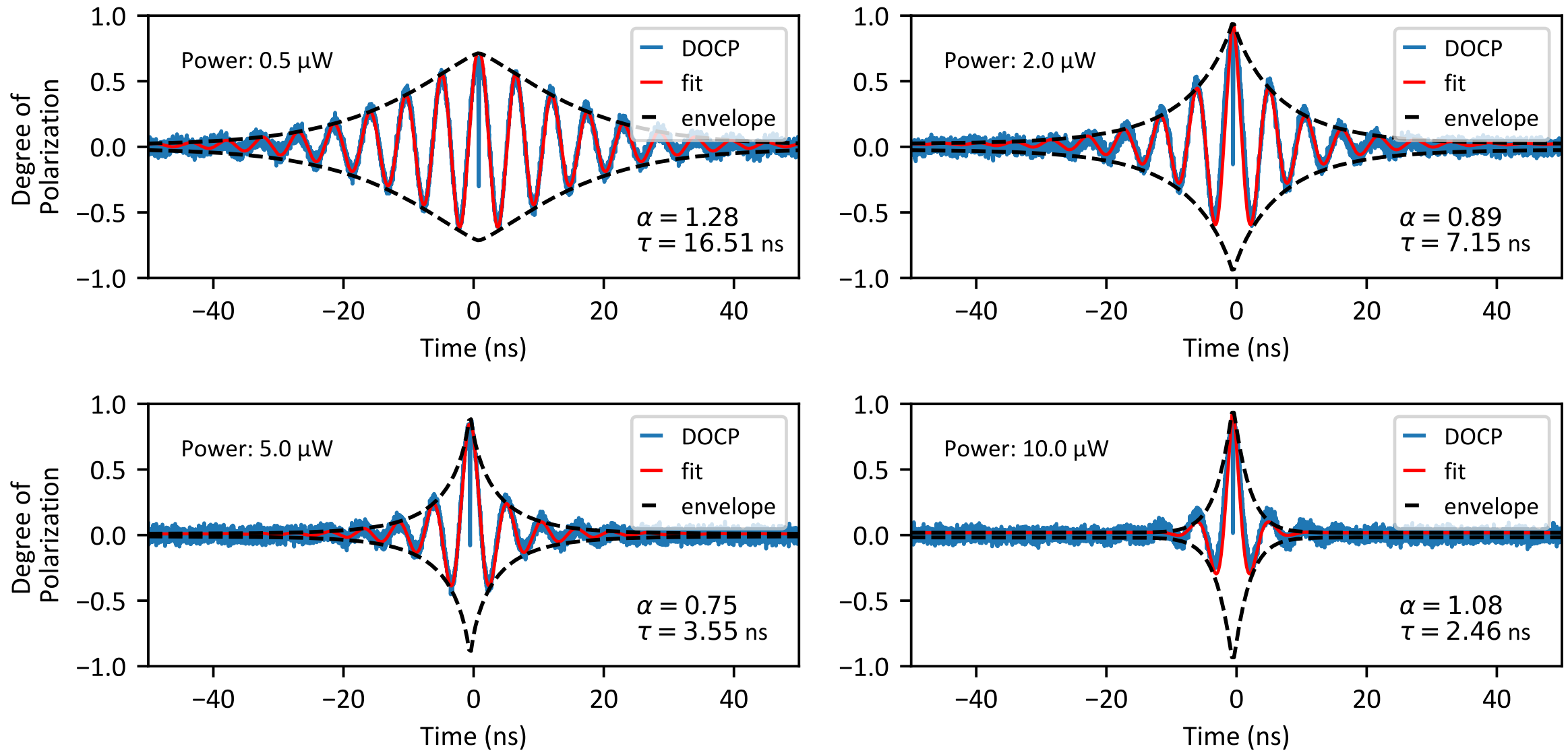

Figure S12: Power series of cw-g2 using circularly polarized excitation. The power is increased from the upper left column to the lower right column. For each power, RR and RL datasets were acquired and the DOCP was calculated and fitted, extracting the dephasing time from the exponentially decaying envelope.

S3.4 Discussion of extracted parameter alpha

In addition to the quasi-static Overhauser field, which has an approximately Gaussian distribution [1], there are several other noise sources that the spin qubits in QDs can couple to, including phonons, charge fluctuations, and electrical noise [2, 3]. Because a hole - in contrast to an electron - couples only weakly to the nuclear bath, these non-nuclear channels can significantly influence the envelope of the dephasing. As a result, fixing $\alpha = 2$ would implicitly assume that nuclear quasi-static noise is the only dominant mechanism, which is not generally valid under our experimental conditions. Under continuous driving, additional fast and non-stationary noise channels become increasingly important: phonon-assisted processes, laser-induced charge trapping, and electric-field–induced g-factor fluctuations, which grow in importance with increasing magnetic field. The coexistence of multiple noise sources with different correlation times naturally leads to $\alpha < 2$.

Based on these considerations and the fitted values of α from cw-g2 measurements varying the laser power and magnetic field (cf. S3.3, S3.1) we chose to set $\alpha = 1$ when fitting Eq. 3 to the data presented in Fig. 3.

S4 Additional information on analysis of pulsed T_2^* measurement

This section provides background data that add to the contents of the pulsed decoherence measurements that are shown in Fig. 3 of the main text.

Spin dephasing and precession frequency depending on readout-time

Fig. S13 shows a plot of the dephasing times and frequencies of the fitted $\langle S_z \rangle$ from the pulsed measurement for different readout times. The vertical red lines mark the boundaries that we chose to average between in order to determine the value of $T_2^* = (15.9 \pm 1.7)$ ns for the hole spin dephasing time.

These boundaries are chosen as follows: Fitting the time traces that are plotted in main text figure Fig. 3 (c) yields the photon arrival time of the second photon t_{pulse2} in both polarizations. Due to the system response time σ, maximum DOCP signal is reached at the point $t_{pulse2} + \sigma$, which we chose as a left boundary. The right boundary is chosen to $t_{pulse2} + \sigma + 220\,\text{ps}$ in order to integrate over one full precession period of the excited state in an in-plane field of 150 mT.

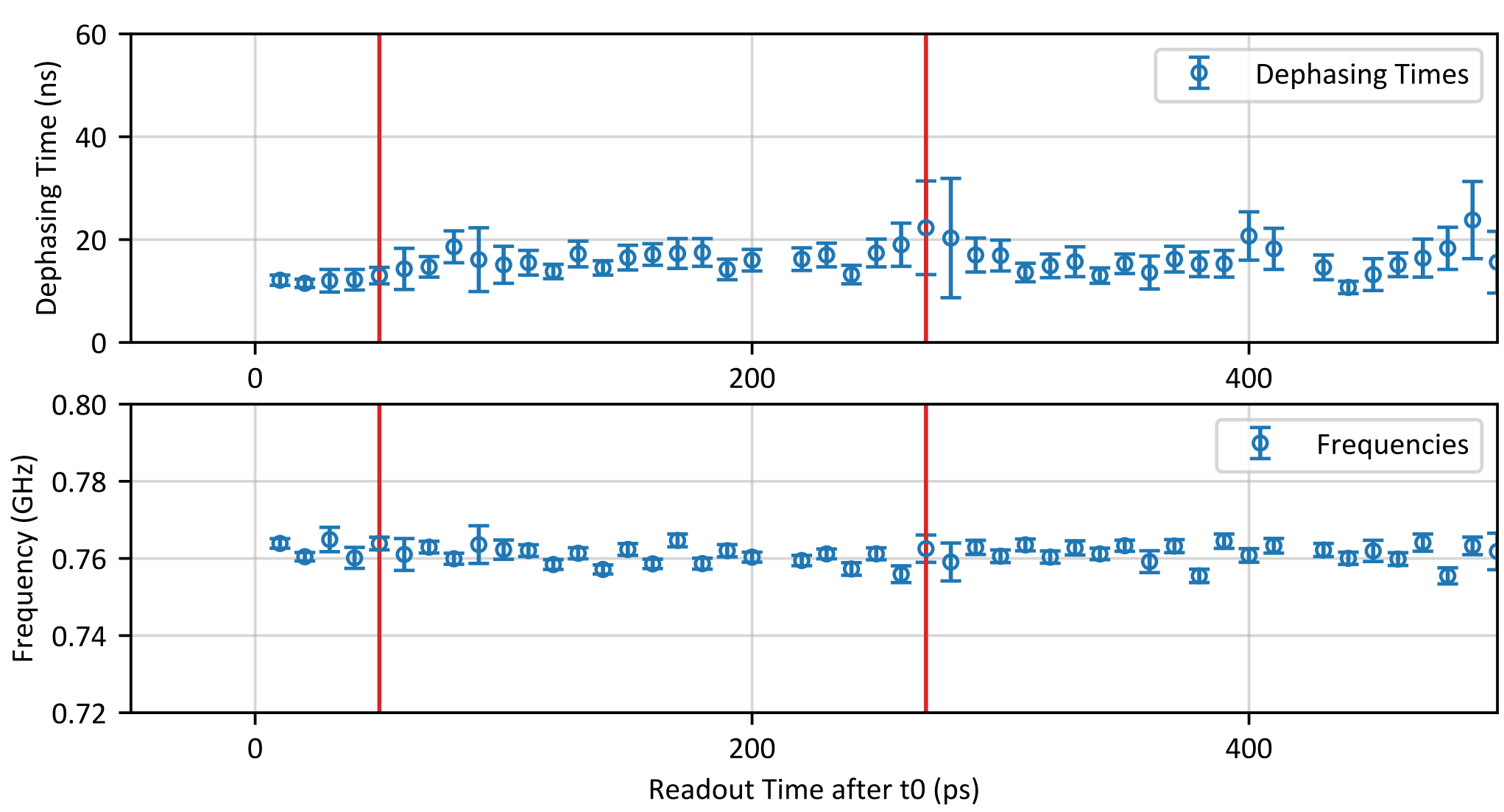

Figure S13: τ and f vs. readout time after t_0. The red vertical lines mark the start and end point, used for averaging over τ and f. The left value is chosen at 50 ps after t_0 to start at the point of maximum PL emission. The right value is chosen at $t_{readout} = 270$ in order to average over one full precession period of the excited state (220 ps at $B_x = 150\,\text{mT}$). Averaging over the dephasing times yields $T_2^* = (15.9 \pm 1.7)$ ns and averaging over f yields $f = 0.761 \pm 0.002$ GHz.

The choice of 220 ps seems natural, since this value approximately agrees with the radiative lifetime of 230 ps measured for the X^+ under quasi-resonant excitation. Even though an even broader integration window could be chosen, extending the right boundary too much becomes impractical once the extracted DOCP is calculated from PL signal close to the noise level. In Fig. S13 this becomes apparent by the increasing errorbars that are adding to the DOCP values for $t > 400\,\mathrm{ps}$.

Nevertheless, we believe that averaging is necessary, since the fitted dephasing times as well as fitted frequencies undergo a certain fluctuation depending on the starting phase of the DOCP fit (cf. main text Fig. 3 (d)). This becomes apparent in both top and bottom panel of Fig. S13 - extracted dephasing times are ranging between (15–20) ns, depending on the readout time, whereas extracted frequencies vary with 2 MHz around the average value.

S5 Magnetic field configurations and selection rules

In the absence of an external magnetic field, the spin quantization axis is defined by the quantum-dot growth direction (taken as the z-axis, which also coincides with the optical axis). The hole in the ground state has a total angular momentum projection of $M_z = \pm\frac{3}{2}$ onto its quantizaiton axis, as it is a heavy hole. The positively charged trion with a total angular momentum z-projection of $M_z = \pm\frac{1}{2}$ therefore forms two degenerate circularly polarized optical transitions (right- (R) and left-circular (L) polarized) to the ground-state hole, following the required spin change of ± 1. R(L) polarized light selectively couples to opposite-sign hole-spin states, providing the natural optical basis for spin initialization and readout.

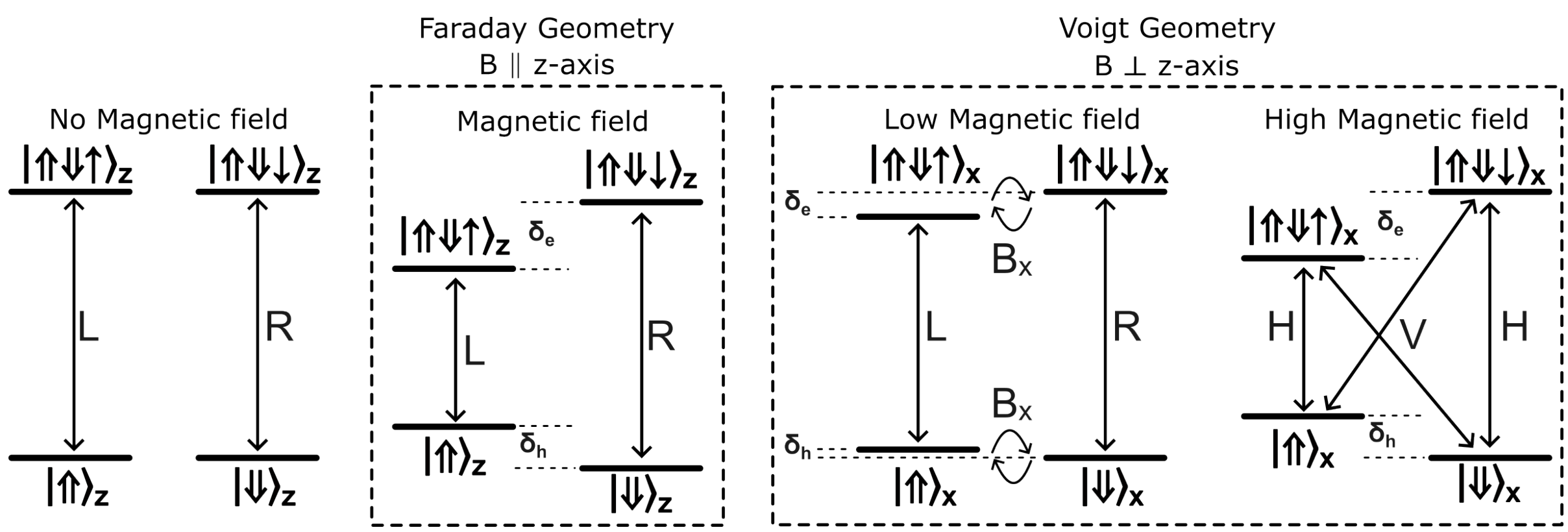

Figure S14: Optical selection rules and spin eigenstates of a positively charged trion under different magnetic-field configurations. Left: zero magnetic field, where the spin quantization axis is defined by the growth (optical) axis and the optical transitions are circularly polarized (R/L). Middle: Faraday geometry ($B \parallel z$), where the Zeeman interaction lifts the spin degeneracy while preserving the spin quantization axis and the circular selection riles. Right: Voigt geometry ($B \perp z$). At low magnetic fields the Zeeman splitting remains smaller than the optical linewidth and the transitions are not spectrally resolved, while coherent spin precession gives rise to polarization oscillations. At sufficiently high magnetic fields the eigenstates becomes aligned with the magnetic field and the four transitions form a spectrally resolved double Λ system with predominantly linear polarization.

Applying a magnetic field parallel to the growth axis (Faraday geometry) lifts the spin degeneracy through the Zeeman interaction while preserving the spin quantization axis. Consequently, the optical selection rules remain circularly polarized and only spin-conserving transitions are allowed. Since optical initialization prepares one of the Zeeman eigenstates, no Larmor precession is expected following initialization unless an additional coherent rotation is applied. This configuration is therefore particularly well suited for spin initialization and high-fidelity optical

readout based on cycling transitions[4], but it is not naturally suited for observing coherent spin precession through polarization oscillations.

In contrast, an in-plane magnetic field (Voigt geometry) rotates the spin quantization axis from the optical axis to the magnetic-field direction. The ground and trion eigenstates become coherent superpositions of the zero-field spin states, resulting in four optically allowed transitions forming a double-Λ system.[5] At sufficiently large magnetic fields the Zeeman splitting exceeds the optical linewidth, allowing the four predominantly linearly polarized (H/V) transitions to be spectrally resolved. In the low-field regime investigated here, however, the Zeeman splitting remains smaller than the optical linewidth, so the transitions are not spectrally resolved and the optical response remains predominantly circularly polarized as the confinement is the dominant energy scale and still defines the eigenstates of the system. Nevertheless, the transverse magnetic field governs the spin dynamics. As a result, the spin state heralded by the first emitted photon is not an eigenstate of the spin Hamiltonian and therefore undergoes coherent Larmor precession about the magnetic field. This coherent evolution is transferred to the polarization of the subsequently emitted photon, producing the oscillatory DOCP signal from which the hole-spin dephasing time is extracted.

The present work exploits this low-field regime, where the spin evolution can be inferred from polarization-resolved photon correlations without requiring spectrally resolved Zeeman transitions. The transition from predominantly circular to predominantly linear polarization is gradual and is governed by the ratio between the Zeeman splitting and the optical linewidth rather than by a sharply defined magnetic field.

Fig. S14 summarizes the optical selection rules of the positively charged Trion in the absence of a magnetic field, in the Faraday geometry, and in the Voigt geometry for both low and high magnetic fields. Here, R/L denotes right- and left-circularly polarized transitions, whereas H/V denotes linearly polarized transitions.

References

[1] I. A. Merkulov, A. L. Efros, *et al.* Electron Spin Relaxation by Nuclei in Semiconductor Quantum Dots. *Physical Review B* **65** (2002), p. 205309.

[2] K. de Greve, P. L. McMahon, *et al.* Ultrafast Coherent Control and Suppressed Nuclear Feedback of a Single Quantum Dot Hole Qubit. *Nature Physics* **7** (2011), pp. 872–878.

[3] J. H. Prechtel, A. V. Kuhlmann, *et al.* Decoupling a Hole Spin Qubit from the Nuclear Spins. *Nature Materials* **15** (2016), pp. 981–986.

[4] J. Dreiser, M. Atatüre, *et al.* Optical investigations of quantum dot spin dynamics as a function of external electric and magnetic fields. *Physical Review B* **77** (**7**) (Feb. 2008), p. 075317.

[5] D. Press, T. D. Ladd, *et al.* Complete quantum control of a single quantum dot spin using ultrafast optical pulses. *Nature* **456** (**7219**) (Jan. 2008). Num Pages: 4, pp. 218–21.
