# Supplementary material for: A spin-photon interface in the telecom C-band with long hole spin dephasing time
Source: Nat Commun. 2026 Sep 2;17:10458. doi: 10.1038/s41467-026-77282-w (PMC13635138; doi:10.1038/s41467-026-77282-w)
Supplement: Supplementary file 2 [file 41467_2026_77282_MOESM2_ESM.pdf]

nature portfolio

Peer Review File

A Spin-Photon Interface in the Telecom C-Band with Long Hole Spin Dephasing Time

Corresponding Author: Mr Johannes Michl

This file contains all reviewer reports in order by version, followed by all author rebuttals in order by version.

Version 0:

Reviewer comments:

Reviewer #1

(Remarks to the Author)
The manuscript A Spin-Photon Interface in the Telecom C-Band with Long Hole Spin Dephasing Time by J. M. Michl et al. addresses an important bottleneck for quantum networking and distributed quantum computing: realizing long-lived, optically addressable spin qubits in telecom-band (1.55 μm) quantum dots. Their progress in telecom quantum dot growth has trailed the near-infrared platform. The authors report deterministically integrated InAs/InAlGaAs quantum dots in a circular Bragg grating emitter (which was reported previously) and provide a clear experimental characterization of the system's spin–photon interface (new). They extract electron and hole g-factors via polarization-resolved spectroscopy of a positively charged trion in an in-plane magnetic field, and they probe the ground-state hole spin dynamics using a pulsed two-photon correlation/ heralding approach, yielding quite long inhomogeneous dephasing. Overall, the work is well written, technically solid, and represents a meaningful step forward in bringing telecom quantum dots to a comparable level of spin-physics maturity as their near-infrared counterparts. Not every part which leads to these great results is completely new. The growth and the CBG fabrication were reported previously (by the same authors) as well as several publications from others have investigated the spins in telecom quantum dot. Still this paper marks an important milestone combining several important technologies together with excellent spectroscopy. Thus, I would still argue for publication for Nature Communication after the authors have convincingly addressed the following points:

- It seems that the general opinion is that Indium degrades the spin coherence time significantly. I would argue that the authors should either give good insights into why this is not the case or discuss how things can be improved in the future. This would fit in the discussion part (somewhere here: P12,L251)

- The paper would benefit from a smoking gun experiment. There are several options the authors clear next steps could be. For me it is not a must, but if referees argue about novelty I suggest to add a spin-photon tomography or similar.

- Can the authors comment on the prospect of using resonant or LA phonon excitation compared to p-shell?

- The authors could give a more detailed explanation on the power induced dephasing and how this might be related to their specific quantum dot structure. (see figure 2)

- I enjoy the cleanliness of Figures. Especially Fig. 3: No excessive use of 3D rendering and very easy to follow. I would think that for early carrier students a setup image in the supplemental would greatly help to educate, given your clear figure style. The supplemental figures S4, S5, and S6 lack behind your other figures. Labels are too small and insets are unreadable.

- P3,L68: Order of references: [33, 34, 31, 32]. Reference 32 now published (update reference)

- And quite some self-citations. I agree that this group is world-leading in telecom growth and optimized it, so I am ok with a bit more self-citations as normal but several papers as references to make the same point is maybe a bit excessive.

- P3,L70 "Only recently, 71 slightly higher values <5 ns could be reported" Please update with numbers from the stimulated arxiv submission 2 days after yours.

- Figure 1. A-c): Please do not use Red and Green for lines. This makes it very difficult for some of us to differentiate the H and V.

- P6,L129: “deviation from CBG ideal position” The authors might want to refer to (their own) work on the displacement and explain in more detail how the CBG is affecting the DOCP. Does this make the initialization harder to detect/herald or does it actually also affect the physics due to other selection rules?

- P7,L157: “Hanbury Brown-Twiss-like” I think you should use a \-- between the surnames. Also what is different in your setup to a standard HBT, that you need to add “-like”.

- Figure 2 a: it would be nice to have a zoom in or a graph in the supplementary (just add it to Fig. S5) showing the dip at tau=0 with better time resolution. I guess the minimal value is limited by the time resolution of your detectors?

- Fig. S7 could need a longer description. Why is this measurement important and how does it compare to others. It seems that the next precession cycle does not influence the measurements (frequency and dephasing seems to be constant). The authors therefore could also have chosen a different integration time. Maybe this can be discussed in more detail.

Reviewer #2

(Remarks to the Author)
Recommendation: Revision

General Comments:

The authors present a well-executed study demonstrating a record inhomogeneous dephasing time of T2* approximately 16 ns for a hole spin in a telecom C-band quantum dot, a meaningful improvement over prior reports in this wavelength range. The measurements are internally consistent, the methodology is sound, and the systematic characterization of power and magnetic field dependences is thorough. However, to fully meet the high-impact threshold expected for this journal, the manuscript must be elevated beyond reporting a purely quantitative improvement. While the reported T2* is highly valuable to the specialized community, the current framing reads as a benchmarking exercise rather than a qualitative breakthrough (such as spin-photon entanglement, cluster state generation, or dynamical decoupling at telecom wavelengths).
The most significant and commendable aspect of this manuscript is the experimental demonstration of this novel DOCP measurement technique to probe the unperturbed spin evolution at low magnetic fields. Currently, the manuscript frames its primary contribution around the quantitative increase of the T2* value for telecom QDs. Relying primarily on this quantitative increase weakens the paper, as it comes across as an empirical observation lacking deep theoretical exploration. To meet the criteria for publication, the authors must shift the emphasis onto the physical implications, theoretical bounds, and broader applicability of their methodology.

I am supportive of publication, but substantial revisions are needed to address the concerns below.

1- Quantitative contribution vs. methodological contribution is not cleanly separated

2- DOCP "approaching NIR" claim needs theory/limits and careful wording
Line 124 / Fig. 1(d) bottom panel: The text states DOCP (86.5 +/- 0.1)% "approaches" NIR values (~95% under LA-phonon assisted excitation). This feels overstated and invites questions:
What is the theoretical ceiling for DOCP in this system under the given quasi-excitation scheme? is there a fundamental bound (<100%) due to heavy-/light-hole mixing, spin-flip probabilities during relaxation, selection-rule leakage, re-excitation, or cavity-induced polarization effects?

3- The manuscript would be significantly stronger if the authors discuss, at least conceptually and practically, the following:
- How would this coherent DOCP-based measurement work under resonant excitation? Resonant excitation can change the initialization pathway, re-excitation probability, and polarization selection dynamics. Would the protocol be practical under the cross-polarized resonance fluorescence detection?

- Compatibility with spin-cooling and "pre-pulse" sequences: Many experiments use sequences (optical pumping, dynamical decoupling preparation, nuclear spin narrowing, etc.) prior to the measurement window. Can the authors clarify: If applied cooling pulses before the "herald" step, can be still used the DOCP oscillation of the emitted photons to extract T2*?
- Differences, limits compared to T2* measurement with traditional Ramsey?

4- Line 265: "reasonable to assume an optimal magnetic field B_opt for which T2* exceeds 17 ns plan to investigate further..."
This reads speculative and should be revised. Unless the authors provide data or a model predicting a maximum at intermediate field, they should either, remove the speculation or provide evidence (physical mechanism, or preliminary points) that plausibly supports an optimum.
Furthermore, relegating the search for the optimal magnetic field to future work is a missed opportunity. Because the quantitative increase in T2* is a central claim of the paper, conducting a field-dependent study to actually find this B_opt would significantly strengthen the manuscript, rather than leaving it as an unverified assumption.

5- Magnetic Field Geometry

(To Supplementary) A dedicated discussion comparing the dynamics, selection rules, and implications of the Voigt geometry versus the Faraday geometry should be added to provide a complete and contextualized picture of the spin-photon interface.

Specific line-by-line comments and requested edits:
Line 77: The authors claim "record values of two-photon interference visibility". The use of the word "record" should be avoided or heavily qualified. Claims of "records" age poorly and can be highly subjective depending on the specific constraints of the material system being compared.

Line 129: "using L (R)-polarized laser illumination". Consider replacing "illumination" with "excitation" or "driving" (more standard for resonant/quasi-resonant optical preparation).

Conclusion:
The manuscript presents a highly valuable experimental achievement, but its full potential is currently limited by a narrative focused primarily on benchmarking T2*. By pivoting the focus toward a rigorous physical exploration of their novel DOCP measurement protocol, clearly defining its theoretical and practical bounds, exploring its operational limits with other excitation regimes and spin species, and replacing speculative assumptions with a concrete approach, the authors can significantly elevate the impact of this work.

Reviewer #3

(Remarks to the Author)
In this manuscript, the authors report on the measurement of the coherence time T2* of a single hole spin in an InAs/InGaAs quantum dot emitting at telecom wavelength. By using time-resolved polarization-resolved correlations of the photons emitted by a positive trion, they infer the coherence time from the decay of the Larmor oscillations in a transverse magnetic field of 150 mT. By using either low power cw excitation or pulsed excitation, they extract a coherence time of about 16ns for a hole spin which is presumably due to the weak hyperfine coupling with the surrounding nuclear spins. This result is consistent with similar semiconductor quantum dots emitting around 900nm. Even though this is likely the first measurement of hole spin T2* for QDs at telecom wavelength and the experimental work sounds to be of high quality, in my opinion there is no strong reason for publication in a high impact journal as Nature Communications. Indeed, the observed T2* time is not really surprising for such QDs, and this investigation sounds quite similar to those presented in Refs. 42 or 43. that were published in more specialized journals. In a revised version, the authors should consider the following points with a specific attention to point 1.

1-The results presented in figure 1.d are extremely puzzling : the polarization of the positive trion under R or L polarized excitation reveals a high degree of circular polarization (DOCP) in zero field and Larmor oscillations in transverse magnetic field during the trion lifetime with a DOCP which apparently does not vanish. For a positive trion (where the spin property corresponds to the electron spin), the hyperfine interaction with the nuclear spins is typically one order of magnitude larger than for the hole spin and a coherence time T2* less than 2ns is expected. Even in zero field, the model of Ref. 48 would lead to fast decay of the polarization during the lifetime with a specific profile (see e.g. Ref.46). A non-vanishing high DOCP at zero field can still be observed under circularly polarized P-shell excitation due to dynamical nuclear polarization (DNP) which can generate an Overhauser field along the optical axis larger than its transverse fluctuations (see Urbaszek et al. Rev. Mod. Phys. 85, 79 (2013)). However, in this case the Larmor oscillations in a transverse magnetic field should be perturbed in a weak magnetic field because of the so-called anomalous Hanle effect (see again Rev. Mod. Phys. 85, 79 (2013)). Quite generally, to investigate the spin dynamics in such quantum dots, excitation with a constant circular polarization can lead to many spurious effect due to DNP and should be avoided. Since it was not specified by the authors, I assume they were not using an excitation with circular polarization alternating at high frequency (above 50kHz) between R and L. Even in this case, the contrast of the oscillations shown in Fig 1d, should significantly vanish in less than 2ns because of the electron spin decoherence. In brief, the dynamics shown in figure 1d is totally unexpected for a positive trion. (Note that in Ref. 42 where the hole spin dynamics is investigated by similar techniques, the authors consider either a positive trion or a negative trion to address the hole spin either in the QD ground state or in the excited state.) In brief, I would recommend the author to further analyze the results of fig.1d, where there is maybe some new physics that could deserve publication in a high impact journal as Nature Communications.

2-Related to main point 1 above, it appears that the authors also use a circularly polarized excitation to investigate the hole spin dynamics in the ground state. This is again surprising, because as shown in Refs. 42 and 43, linearly polarized excitation works as well and is likely preferable to avoid spurious effects like DNP. The authors should further comment on this choice of circular polarized excitation rather than linear polarization.

3- The manuscript could also benefit from an improved discussion on the empirical model (Eq. (3)) used to fit the data because the parameter alpha close to unity (and showing significant variations against power) does not correspond to the Gaussian distribution of Overhauser fields (alpha=2) which should be considered according to Ref. 48. This could indicate other sources of magnetic noise for these QDs.

4- The power dependence shown in Fig.2d is fitted as an exponential decay but this decay of dephasing time at high power is likely due to the shortening of the lifetime in the ground state (due to faster excitation) for which a simple model would likely give a dephasing time proportional to 1/(1/T2* + eta P^0.5) where P is the power and eta a fitting parameter.

Version 1:

Reviewer comments:

Reviewer #1

(Remarks to the Author)
The revised manuscript “A Spin-Photon Interface in the Telecom C-Band with Long Hole Spin Dephasing Time” by J. M. Michl et al. has improved significantly.
All my comments have been addressed, and I appreciate the detailed added discussions. In Particular, the point raised by reviewer 3 about the possible creation of unwanted DNP due to the circular polarization was very important.
For me the manuscript gives enough insights and advances our understanding that another smoking gun experiment is not needed. But I can understand if other reviewers see this differently.

Reviewer #2

(Remarks to the Author)
The authors have satisfactorily addressed all of my comments. The manuscript has been substantially improved in clarity and overall quality. I therefore recommend the manuscript for publication in Nature Communications.

Reviewer #3

(Remarks to the Author)
The authors have satisfactorily taken into account the criticisms I raised in my first review, notably by performing new measurements with different configuration of excitation polarization, and by extracting from their data the trion (i.e. electron) spin coherence of 1.2 ns. The main result of this study regarding the hole spin coherence of about 20ns in telecom QDs is quite convincing. Even though in my opinion this result is not really surprising, this type of research on telecom QDs is topical and the correlation-based measurements nicely illustrate the maturity of the technology which turns out very encouraging for future demonstration of multi-entangled photons at telecom wavelength. I therefore recommend publication in Nature Communication after a few (not totally minor) corrections/revisions.

1- The abstract does not reflect an important conclusion of the study that the spin coherence time depends significantly on the magnetic field in the 0-150 mT range, and that the measurements with pulsed excitation need to be extended to lower magnetic fields to confirm this trend.

2- In fig 1b, the color code for H/V transitions should be swapped to be consistent with results in fig 1a.

3- l.243 : the claim that pulse 1 initializes the spin is misleading... This is the detection in R or L polarization of the photon emitted after pulse 1 which effectively performs a projective measurement, and thus a kind of initialization. (As for the measurements in cw regime, the author could have used any linear polarization for pulse 1, which would suppress the oscillations along the vertical axis in fig 3b, while keeping the oscillations along the horizontal axis).

4- Using correlations of photons emitted under pulse excitation is not really a "novel approach" to measure the T2*, since to some extent, it corresponds to a simpler version of the Lindner-Rudolph protocol which has been implemented by several teams to demonstrate cluster states with NIR quantum dots (e.g. Ref. 20 and 21 or more specifically Coste et al., Nat. Photonics 17, 582 (2023))). The authors should moderate the corresponding claim when they introduce the technique as well as in the conclusion.

5- There is no information about the time resolution/jitter of the detectors. This likely explains the reduced contrast of DOCP oscillations in fig.1 as compared to the spin conservation fidelity in zero field.

6- While I agree that the measurements reported in Fig. S6 show no sign of DNP (the period of electron spin precession seems to be exactly the same in both configurations), the observation of a strong increase of DOCP under cw circular excitation reported in Fig. S10b could be in contrast interpreted as a reduction of the electron spin precession frequency due to DNP. The explanation that this increase is due to "deterministic re-initialization of the trion state in its $|T\uparrow\rangle$ ($|T\downarrow\rangle$) state through the L(R) polarized laser" sounds to me quite incorrect. The dependence of initial DOCP (namely just after hole spin initialization) on magnetic field is mostly governed by the trion spin precession during the lifetime (Hanle effect). Under H-polarized excitation it shows a Lorentzian-like decay above 10mT (as expected when there is no DNP), while under circular polarization the DOCP remains almost constant at a very high level as if the magnetic field experienced by the electron spin is strongly reduced due to DNP effect. The authors should be more careful when commenting the results of Fig. S10.

Open Access This Peer Review File is licensed under a Creative Commons Attribution 4.0 International License, which permits use, sharing, adaptation, distribution and reproduction in any medium or format, as long as you give appropriate credit to the original author(s) and the source, provide a link to the Creative Commons license, and indicate if changes were made.
In cases where reviewers are anonymous, credit should be given to 'Anonymous Referee' and the source.
The images or other third party material in this Peer Review File are included in the article's Creative Commons license, unless indicated otherwise in a credit line to the material. If material is not included in the article's Creative Commons license and your intended use is not permitted by statutory regulation or exceeds the permitted use, you will need to obtain permission directly from the copyright holder.
To view a copy of this license, visit https://creativecommons.org/licenses/by/4.0/

Manuscript: A Spin-Photon Interface in the Telecom C-Band with Long Hole Spin Dephasing Time

REVIEWER COMMENTS

Reviewer #1 (Remarks to the Author):

The manuscript A Spin-Photon Interface in the Telecom C-Band with Long Hole Spin Dephasing Time by J. M. Michl et al. addresses an important bottleneck for quantum networking and distributed quantum computing: realizing long-lived, optically addressable spin qubits in telecom-band (1.55 μm) quantum dots. Their progress in telecom quantum dot growth has trailed the near infrared platform. The authors report deterministically integrated InAs/InAlGaAs quantum dots in a circular Bragg grating emitter (which was reported previously) and provide a clear experimental characterization of the system's spin–photon interface (new). They extract electron and hole g-factors via polarization-resolved spectroscopy of a positively charged trion in an in-plane magnetic field, and they probe the ground-state hole spin dynamics using a pulsed two-photon correlation/ heralding approach, yielding quite long inhomogeneous dephasing. Overall, the work is well written, technically solid, and represents a meaningful step forward in bringing telecom quantum dots to a comparable level of spin-physics maturity as their near infrared counterparts. Not every part which leads to these great results is completely new. The growth and the CBG fabrication were reported previously (by the same authors) as well as several publications from others have investigated the spins in telecom quantum dot. Still this paper marks an important milestone combining several important technologies together with excellent spectroscopy. Thus, I would still argue for publication for Nature Communication after the authors have convincingly addressed the following points:

- It seems that the general opinion is that Indium degrades the spin coherence time significantly. I would argue that the authors should either give good insights into why this is not the case or discuss how things can be improved in the future. This would fit in the discussion part (somewhere here: P12,L251)

Author response: We agree with the reviewer that this is the dominant opinion in the community. To address this point, we added a discussion in the manuscript (Pages 13-14, Lines 283-303 of track change document) clarifying why long hole spin dephasing time can still be achieved in Indium based quantum dots. We additionally outline the possible next steps to further improvement.

- The paper would benefit from a smoking gun experiment. There are several options the authors clear next steps could be. For me it is not a must, but if referees argue about novelty I suggest to add a spin-photon tomography or similar.

Author response: While we agree with the reviewer that a spin-photon tomography is a nice experiment, it is not the scope of the manuscript to investigate spin-photon entanglement dynamics, which would take the focus away from the presented results. We do believe that the presented spin dynamics and methods to measure them are a valuable contribution by itself.

- Can the authors comment on the prospect of using resonant or LA phonon excitation compared to p-shell?

Author response: We chose p-shell excitation because of its simple experimental realization and robust spin selective excitation. However, relaxation through higher energy states can introduce spin non-conserving processes. Resonant excitation as well as LA phonon assisted avoids the relaxation cascade entirely and is therefore expected to improve spin initialization fidelity. However, we do not think that the chosen excitation scheme would influence the ground state spin dynamics. It would only change the initialization fidelity, thus it would not reveal different physics. We included a measurement of spin initialization using LA phonon assisted excitation in the SI (page 8 of SI, section S2.3, Fig. S7) to show that it indeed improves the DOCP from a maximum value of 0.92 (p-shell) to 0.97 (LA phonon).

- The authors could give a more detailed explanation on the power induced dephasing and how this might be related to their specific quantum dot structure. (see figure 2)

Author response: We thank the reviewer for this valuable comment. A detailed discussion can be found below in our response to Reviewer 3 (Comment 4). Following the reviewer's suggestion, we reanalyzed the power dependence using a phenomenological model in which the dephasing rate increases with excitation power. The revised analysis shows that the measured T2* is well described by an inverse linear dependence, $T_2^*(P) = 1/(m^*P + b)$, consistent with an additional excitation-induced decoherence channel over the investigated power range. One possible microscopic origin is an additional excitation event occurring before completion of the free spin evolution, effectively shortening the available ground-state evolution time. While structure-dependent effects may contribute to the overall spin dephasing, the present analysis indicates that they are not required to account for the observed power dependence. We have added this discussion to the revised manuscript (Page 9, Lines 199-205 of track change document).

- I enjoy the cleanliness of Figures. Especially Fig. 3: No excessive use of 3D rendering and very easy to follow. I would think that for early carrier students a setup image in the supplemental would greatly help to educate, given your clear figure style. The supplemental figures S4, S5, and S6 lack behind your other figures. Labels are too small and insets are unreadable.

Author response: We thank the reviewer for this compliment. We modified the figures in the SI to have readable labels and insets. Additionally, we have included a sketch of the setup in the supplement (figure S4, page 5 of SI, section S1.4) since we agree that providing visual illustration of the setup can help interested readers that are not familiar with complex optical setups to gain more insight to the topic.

- P3,L68: Order of references: [33, 34, 31, 32]. Reference 32 now published (update reference)

Author response: We thank the reviewer for pointing out this error. We updated our latex code to automatically sort correctly.

- And quite some self-citations. I agree that this group is world-leading in telecom growth and optimized it, so I am ok with a bit more self-citations as normal but several papers as references to make the same point is maybe a bit excessive.

Author response: We thank the reviewer for this comment. We agree that citation clusters should not be used to support the same point redundantly, and we have therefore carefully reviewed the citation structure throughout the manuscript.

Our previous work is cited where it documents distinct steps in the development of the telecom quantum-dot platform, including growth optimization, device integration, excitation schemes, and spin control. At the same time, we recognize that some citation clusters could give the impression of being overly weighted toward our own prior work. We have therefore made targeted changes to reduce redundancy and improve the balance of the references.

Specifically, in the Introduction, in the sentence discussing improved optical properties due to refined growth conditions, we reduced the number of references from three to two by removing one citation that was not essential for this specific point. In the indistinguishability section, we removed one internal reference while retaining external and collaborative works, including Joos 2024, Holewa 2024, and Hauser 2026, so that the citation cluster now better reflects the broader literature. We also cite Behrends et al. 2026, a collaborative work with the Heindel group, where it provides relevant evidence for the excitation-scheme dependence on samples from this material platform.

We hope that these targeted changes address the reviewer's concern and make the use of references more balanced and non-redundant.

- P3,L70 "Only recently, 71 slightly higher values <5 ns could be reported" Please update with numbers from the stimulated arxiv submission 2 days after yours.

Author response: We have updated the sentence to "[...] slightly higher values ≤ 7 ns could be reported." to correctly include the mentioned arxiv submission that appeared after our initial submission. The change appears in line 72, page 3 of the track change document.

- Figure 1. A-c): Please do not use Red and Green for lines. This makes it very difficult for some of us to differentiate the H and V.

Author response: We thank the reviewer for bringing this to our attention. We have selected a different color palette that brings more contrast for people with difficulties in distinguishing red and green.

- P6,L129: "deviation from CBG ideal position" The authors might want to refer to (their own) work on the displacement and explain in more detail how the CBG is affecting the DOCP. Does this make the initialization harder to detect/herald or does it actually also affect the physics due to other selection rules?

Author response: We thank the reviewer for pointing out that this was not well explained. We measured the DOLP for this device after this comment and see no measurable influence from the

CBG, as the QD is placed centered (to within the errors of our DOLP measurement). Since the eigenmodes of the CBG are linear, DOLP different to zero would show a displacement of the QD with respect to the center of the CBG, as also shown in our previous works. In the case of zero DOLP, we also do not expect any influence on the DOCP. We rather believe that the residual deviation from one in the DOCP measurement comes from the excitation scheme, which we tested by a control measurement using LA-phonon assisted excitation (see SI page 8, Fig. S7). We rephrased our statement accordingly and added a more detailed discussion on the underlying mechanisms. The changes appear in lines 126-137 on page 6 of the track change document.

- P7,L157: “Hanbury Brown-Twiss-like” I think you should use a \-- between the surnames. Also what is different in your setup to a standard HBT, that you need to add “-like”.

Author response: We have implemented the changes and decided to go with the convention “Hanbury Brown and Twiss”. The word “like” was only added because publications on standard (non polarization resolved) HBT experiments usually focus on the multi-photon probability at zero time delay for benchmarking their single photon source. In our work, on the other hand, we utilized a polarization resolved HBT to focus on the spin heralding capabilities of the setup. The change appears in line 165, page 7 of the track change document.

- Figure 2 a: it would be nice to have a zoom in or a graph in the supplementary (just add it to Fig. S5) showing the dip at tau=0 with better time resolution. I guess the minimal value is limited by the time resolution of your detectors?

Author response: We have followed the reviewers’ excellent suggestion and have added a graph in the supplementary (Fig. S9, page 11 of SI, section S3.1) that shows zoomed in plots of two different datasets. We have used this chance to include a more detailed description of our data analysis and processing in the description of this figure. For most of the experiments, we chose a bin width of 20 ps which is longer than the resolution of the SNSPDs (timing jitter < 20 ps). Since most of our results that we present in this work are acquired through two-photon correlation measurements, acquisition time per dataset was a relevant issue to consider. We therefore chose to not use the finest bin width possible, although we agree with the reviewer that - in order to perfectly resolve the dip at tau=0 one would need to measure with the best time resolution possible.

- Fig. S7 could need a longer description. Why is this measurement important and how does it compare to others. It seems that the next precession cycle does not influence the measurements (frequency and dephasing seems to be constant). The authors therefore could also have chosen a different integration time. Maybe this can be discussed in more detail.

Author response: We thank the reviewer for pointing out that Fig. S7 (Fig. S13 in the revised manuscript), as well as the corresponding description of our data analysis, required further clarification. We have expanded the discussion in SI section S4 to provide a more detailed description of the analysis procedure, including the reasoning behind the chosen averaging boundaries and why this integration window was selected for determining the reported T_2* value.

Reviewer #2 (Remarks to the Author):

Recommendation: Revision

General Comments:

The authors present a well-executed study demonstrating a record inhomogeneous dephasing time of T2* approximately 16 ns for a hole spin in a telecom C-band quantum dot, a meaningful improvement over prior reports in this wavelength range. The measurements are internally consistent, the methodology is sound, and the systematic characterization of power and magnetic field dependences is thorough. However, to fully meet the high-impact threshold expected for this journal, the manuscript must be elevated beyond reporting a purely quantitative improvement. While the reported T2* is highly valuable to the specialized community, the current framing reads as a benchmarking exercise rather than a qualitative breakthrough (such as spin-photon entanglement, cluster state generation, or dynamical decoupling at telecom wavelengths).

The most significant and commendable aspect of this manuscript is the experimental demonstration of this novel DOCP measurement technique to probe the unperturbed spin evolution at low magnetic fields. Currently, the manuscript frames its primary contribution around the quantitative increase of the T2* value for telecom QDs. Relying primarily on this quantitative increase weakens the paper, as it comes across as an empirical observation lacking deep theoretical exploration. To meet the criteria for publication, the authors must shift the emphasis onto the physical implications, theoretical bounds, and broader applicability of their methodology.

I am supportive of publication, but substantial revisions are needed to address the concerns below.

1- Quantitative contribution vs. methodological contribution is not cleanly separated

Author response: We thank the reviewer to point out that for him/her it was not clear what was quantitative or what was methodological contribution. The original manuscript P.7 151-155 reads: “In a first experiment we studied the spin under continuous-wave quasi-resonant excitation as has been demonstrated in recent years by various groups (Coste.2023, Serov.2025). Following this, we extend the results by following a different approach that utilizes excitation with two pulses with variable delay and polarization.” We believe that maybe the phrasing “different approach” without citation was not clearly stating new methodology. We thus rephrased to “novel approach”. We hope that this resolves the reviewer’s concern. The change appears in line 162, page 7 of the track change document.

2- DOCP "approaching NIR" claim needs theory/limits and careful wording

Line 124 / Fig. 1(d) bottom panel: The text states DOCP (86.5 +/- 0.1)% "approaches" NIR values (~95% under LA-phonon assisted excitation). This feels overstated and invites questions:

What is the theoretical ceiling for DOCP in this system under the given quasi-excitation scheme? is there a fundamental bound (<100%) due to heavy-/light-hole mixing, spin-flip probabilities during relaxation, selection-rule leakage, re-excitation, or cavity-induced polarization effects?

Author response: The DOCP is mainly limited by heavy-hole–light-hole mixing in the p-shell state, which leads to a selection-rule leakage and spin-flip probability during relaxation. Contributions from possible re-excitations are bound by the measured g(2)<0.01. We tested the assumption that the DOCP is limited by mechanisms caused by our excitation scheme with a control measurement using LA-phonon assisted excitation (see SI Fig. S7), where the DOCP response is closer to the ideal behavior. We do not assign a quantitative theoretical ceiling for the DOCP, since such a number would be highly system- and excitation-scheme-dependent. It would require a microscopic model of the specific quantum dot, including its morphology, composition, strain profile, excited-state valence-band mixing, spin-flip probabilities during relaxation, possible re-excitation, and the polarization response of the photonic environment. Such a model is beyond the scope of the present work and is not available with sufficient accuracy for the investigated emitter. We have therefore added a qualitative discussion of this limitation to the manuscript and explicitly identify the p-shell excitation pathway as the likely origin of the reduced DOCP contrast.

3- The manuscript would be significantly stronger if the authors discuss, at least conceptually and practically, the following:

- How would this coherent DOCP-based measurement work under resonant excitation? Resonant excitation can change the initialization pathway, re-excitation probability, and polarization selection dynamics. Would the protocol be practical under the cross-polarized resonance fluorescence detection?

Author response: Reviewer 1 had basically the exact same question. We reproduce the answer to reviewer 1 here: "We chose p-shell excitation because of its simple experimental realization and robust spin selective excitation. However, relaxation through higher energy states can introduce spin non-conserving processes. Resonant excitation as well as LA phonon assisted avoids the relaxation cascade entirely and is therefore expected to improve spin initialization fidelity. However, we do not think that the chosen excitation scheme would influence the ground state spin dynamics, it would only change the initialization fidelity, thus it would not reveal different physics. We included a measurement of spin initialization using LA phonon assisted excitation in the SI (Fig. S7) to show that it indeed improves the DOCP from a maximum value of 0.92 (p-shell) to 0.97 (LA phonon)."

The protocol can be performed under cross-polarization in resonant excitation. The number of measurements would double, as both the responses in R(L) have to be measured using a different excitation laser polarization L(R) and consequently also the cross-polarization for extinction of the resonant laser would need to be changed for every measurement setting. This does not seem very practical, but it would be in principle possible.

- Compatibility with spin-cooling and "pre-pulse" sequences: Many experiments use sequences (optical pumping, dynamical decoupling preparation, nuclear spin narrowing, etc.) prior to the measurement window. Can the authors clarify: If applied cooling pulses before the "herald" step, can be still used the DOCP oscillation of the emitted photons to extract T2*?

Author response: We thank the reviewer for this insightful question. In the protocol presented here, the spin is initially not prepared deterministically. Instead, detection of the first photon

heralds the preparation of a well-defined spin state, and the second pulse probes the subsequent free evolution of this heralded state. The DOCP oscillations are therefore used to extract (T2*) from the free-induction dynamics after the heralding event.

Preparation sequences applied before the heralding step, such as optical pumping or nuclear-spin narrowing, do not in principle preclude the use of the DOCP method. If an optical pumping sequence already prepares the carrier spin, the role of the heralding photon is modified: it may act more as a verification or post-selection step rather than as the primary spin-initialization step. In this case, the protocol would have to be adapted accordingly, but the subsequent DOCP oscillation could still be used to monitor the spin evolution after a well-defined initialization event.

For nuclear-spin preparation or narrowing, the situation is different. Such a sequence primarily modifies the Overhauser-field distribution experienced by the carrier spin. This would not change the basic principle of the measurement, but it would change the quantity being measured: a narrowed nuclear-spin distribution would be expected to lead to a longer apparent (T2*), since the inhomogeneous dephasing from the fluctuating Overhauser field is reduced.

More complex pre-pulse sequences involving coherent spin rotations or dynamical-decoupling-type control are also not excluded in principle. However, in that case the extracted timescale becomes sequence-dependent and does in general not reveal T2*, but rather a protocol dependent T2 time, unless the interval after the herald is kept as a simple free-evolution interval. We want to note that many preparation protocols work in a different magnetic field regime, i.e. in higher fields, where the ground state splitting can be resolved in frequency.

We added a paragraph to discuss this topic into the Discussion section (page 14 of track change document, lines 306-312)

- Differences, limits compared to T2* measurement with traditional Ramsey?

Author response: Traditional Ramsey interferometry remains the standard approach for measuring T2* because it provides access to coherent spin control through well-defined rotation pulses. In contrast, the DOCP approach presented here extracts T2* from polarization-resolved photon correlations and therefore probes the spin evolution through the spin-photon interface itself.

A key advantage of this method is that it does not require deterministic spin initialization via optical pumping or coherent spin control pulses. Instead, the spin state is prepared through heralding, and the subsequent free evolution is inferred from the polarization of the emitted photons. Consequently, the method can be implemented without identifying and calibrating pi/2 rotation settings, which are mandatory in Ramsey measurements.

At the same time, the present approach does not replace Ramsey interferometry. Ramsey-based techniques provide access to arbitrary spin rotations, coherent control protocols such as dynamical decoupling sequences. By contrast, the DOCP method is primarily designed to characterize spin evolution through optical selection rules and photon-polarization correlations.

We have added a few lines on this topic in the Discussion section (pages 14-15 of track change document, lines 320-325).

4- Line 265: "reasonable to assume an optimal magnetic field B_opt for which T2* exceeds 17 ns plan to investigate further..."

This reads speculative and should be revised. Unless the authors provide data or a model predicting a maximum at intermediate field, they should either, remove the speculation or provide evidence (physical mechanism, or preliminary points) that plausibly supports an optimum.

Furthermore, relegating the search for the optimal magnetic field to future work is a missed opportunity. Because the quantitative increase in T2* is a central claim of the paper, conducting a field-dependent study to actually find this B_opt would significantly strengthen the manuscript, rather than leaving it as an unverified assumption.

Author response: We agree with the reviewer that the sentence was speculative, although based on reasonable assumptions. To verify our speculation, we performed more experiments in the search for the optimal magnetic field and found an even higher value than originally reported. Using linearly polarized excitation and with a magnetic field of B = 20 mT we have found a value of T2* = 21.8 ± 0.2 ns. We revised the sentence in question and added the new dataset that utilizes H-excitation and explores lower magnetic fields using low excitation powers by replacing Fig. 2 of the manuscript. We thank the reviewer for the input since we agree that these additional data significantly strengthen the manuscript. We have revised our reported value of T2* in the abstract to this new best value.

5- Magnetic Field Geometry

(To Supplementary) A dedicated discussion comparing the dynamics, selection rules, and implications of the Voigt geometry versus the Faraday geometry should be added to provide a complete and contextualized picture of the spin-photon interface.

Author response: To address this point, we added a dedicated discussion to the Supplementary Information (section S5), comparing the optical selection rules, spin dynamics, and implications of the Faraday and Voigt geometries.

Specific line-by-line comments and requested edits:

Line 77: The authors claim "record values of two-photon interference visibility". The use of the word "record" should be avoided or heavily qualified. Claims of "records" age poorly and can be highly subjective depending on the specific constraints of the material system being compared.

Author response: We thank the referee for the comment and agree. We exchanged “record” with “new state of the art”.

The change appears in line 78 of the track change document.

Line 129: "using L (R)-polarized laser illumination". Consider replacing "illumination" with "excitation" or "driving" (more standard for resonant/quasi-resonant optical preparation).

Author response: We agree with the referee's comment and have changed the word to "excitation".

The change appears in line 138 on page 6 of the track change document.

Conclusion:

The manuscript presents a highly valuable experimental achievement, but its full potential is currently limited by a narrative focused primarily on benchmarking T2*. By pivoting the focus toward a rigorous physical exploration of their novel DOCP measurement protocol, clearly defining its theoretical and practical bounds, exploring its operational limits with other excitation regimes and spin species, and replacing speculative assumptions with a concrete approach, the authors can significantly elevate the impact of this work.

Author response: We thank the reviewer for judging our manuscript as highly valuable. Besides the above explicit questions, we also addressed the additionally implied questions that the conclusion asks and performed the additional changes: we extracted the T2* for the electron spin and show the data in Fig. 3. We added a discussion on other excitation regimes, and discuss that limited DOCP does not affect the ground state spin physics that the method reveals. We also added a discussion comparing the DOCP protocol with conventional Ramsey interferometry, highlighting both its practical advantages and its limitations. We replaced speculative assumptions and performed control experiments. To conclude, we want to thank the reviewer for making our manuscript better with his/her valuable comments.

Reviewer #3 (Remarks to the Author):

In this manuscript, the authors report on the measurement of the coherence time T2* of a single hole spin in an InAs/InGaAs quantum dot emitting at telecom wavelength. By using time-resolved polarization-resolved correlations of the photons emitted by a positive trion, they infer the coherence time from the decay of the Larmor oscillations in a transverse magnetic field of 150 mT. By using either low power cw excitation or pulsed excitation, they extract a coherence time of about 16ns for a hole spin which is presumably due to the weak hyperfine coupling with the surrounding nuclear spins. This result is consistent with similar semiconductor quantum dots emitting around 900nm. Even though this is likely the first measurement of hole spin T2* for QDs at telecom wavelength and the experimental work sounds to be of high quality, in my opinion there is no strong reason for publication in a high impact journal as Nature Communications. Indeed, the observed T2* time is not really surprising for such QDs, and this investigation sounds quite similar to those presented in Refs. 42 or 43. that were published in more specialized journals. In a revised version, the authors should consider the following points with a specific attention to point 1.

1-The results presented in figure 1.d are extremely puzzling : the polarization of the positive trion under R or L polarized excitation reveals a high degree of circular polarization (DOCP) in zero field and Larmor oscillations in transverse magnetic field during the trion lifetime with a DOCP which apparently does not vanish. For a positive trion (where the spin property corresponds to the electron spin), the hyperfine interaction with the nuclear spins is typically one order of magnitude larger than for the hole spin and a coherence time T2* less than 2ns is expected. Even in zero field, the model of Ref. 48 would lead to fast decay of the polarization during the lifetime with a specific profile (see e.g. Ref.46). A non-vanishing high DOCP at zero field can still be observed under circularly polarized P-shell excitation due to dynamical nuclear polarization (DNP) which can generate an Overhauser field along the optical axis larger than its transverse fluctuations (see Urbaszek et al. Rev. Mod. Phys. 85, 79 (2013)). However, in this case the Larmor oscillations in a transverse magnetic field should be perturbed in a weak magnetic field because of the so-called anomalous Hanle effect (see again Rev. Mod. Phys. 85, 79 (2013)). Quite generally, to investigate the spin dynamics in such quantum dots, excitation with a constant circular polarization can lead to many spurious effect due to DNP and should be avoided. Since it was not specified by the authors, I assume they were not using an excitation with circular polarization alternating at high frequency (above 50kHz) between R and L. Even in this case, the contrast of the oscillations shown in Fig 1d, should significantly vanish in less than 2ns because of the electron spin decoherence. In brief, the dynamics shown in figure 1d is totally unexpected for a positive trion. (Note that in Ref. 42 where the hole spin dynamics is investigated by similar techniques, the authors consider either a positive trion or a negative trion to address the hole spin either in the QD ground state or in the excited state.) In brief, I would recommend the author to further analyze the results of fig.1d, where there is maybe some new physics that could deserve publication in a high impact journal as Nature Communications.

Author response: We thank the reviewer for bringing these well informed concerns about dynamic nuclear polarization to our attention. We agree that the previous manuscript did not sufficiently address these concerns. Therefore, we have conducted extensive additional measurements and are now confident that DNP can be disregarded for the results we present in the revised manuscript.

Firstly - as suggested by the reviewer - we have performed a control experiment that alternates between R and L polarization with high frequency, i.e., every applied pulse is different, and have added the result to the supplement (cf. section S2.2 including Fig. S6). The resulting degree of polarization for alternating polarizations shows no significant deviation from the case where we excite only with R, except a phase flip of 180 degrees that is expected, since we initialize the trion in its orthogonal spin state using L instead of R. Additionally, we have quantified a lower bound for the electron spin dephasing in our quantum dot (cf. Fig. 3 (c)). A fit to the DOCP of the time traces yields an electron spin dephasing time of 1.2(1) ns which is a little more than 5 times the radiative lifetime of the positive trion. Considering this, we think it is not surprising that the DOCP does not vanish within the time window of the trion decay as noticed by the reviewer above. We additionally note that the measurements shown in Fig. 3 employ a linearly H-polarized laser pulse as the second pulse, which does not provide a preferential optical orientation axis and therefore argues against dynamic nuclear polarization in this case.

We hope that the control measurement, together with the additional explanation of the pulsed measurement protocol, satisfactorily addresses the reviewer's concern. The possible role of dynamic nuclear polarization is discussed further in our response to the following comment, where the reviewer elaborates on this point.

2-Related to main point 1 above, it appears that the authors also use a circularly polarized excitation to investigate the hole spin dynamics in the ground state. This is again surprising, because as shown in Refs. 42 and 43, linearly polarized excitation works as well and is likely preferable to avoid spurious effects like DNP. The authors should further comment on this choice of circular polarized excitation rather than linear polarization.

Author response: We thank the reviewer for raising this important point. We agree that linearly polarized excitation is the more appropriate configuration to minimize possible artifacts from optically induced dynamic nuclear polarization. In response to the reviewer's comment, we therefore repeated the measurement of the ground-state hole-spin dynamics using linearly H-polarized excitation. We also used this opportunity to extend the measurement to lower magnetic fields, as suggested by Reviewer 2, in order to identify the magnetic-field range yielding the longest extracted dephasing time.

The revised Fig. 2 now shows the data obtained under linearly H-polarized excitation. A direct comparison with circularly polarized excitation is provided in Supplementary Section S3.2 and Fig. S10. We find that the extracted dephasing times are of the same order for both excitation configurations and decrease with increasing magnetic field in both cases. The linearly excited data yield slightly longer dephasing times, whereas circular excitation gives a larger initial polarization contrast, as expected from its more direct spin-selective initialization of the trion.

We do not interpret the difference between the two excitation configurations as evidence for a specific microscopic mechanism. Instead, we use the linearly excited data for the main-text analysis because this configuration avoids a preferential optical orientation axis and therefore provides the more conservative measurement with respect to possible DNP-related artifacts. The circularly excited data are retained in the Supplementary Information as a comparison and to document the role of spin-selective initialization.

3- The manuscript could also benefit from an improved discussion on the empirical model (Eq. (3)) used to fit the data because the parameter alpha close to unity (and showing significant variations against power) does not correspond to the Gaussian distribution of Overhauser fields (alpha=2) which should be considered according to Ref. 48. This could indicate other sources of magnetic noise for these QDs.

Author response: We thank the reviewer for pointing this out. We agree that a purely quasi-static Gaussian distribution of Overhauser fields would lead to a Gaussian decay envelope, corresponding to alpha=2. However, this limit is most directly applicable when the dephasing is dominated by frozen nuclear-field fluctuations, as is commonly considered for electron spins with strong Fermi-contact hyperfine coupling. In the present case, the measured spin is a ground-state hole spin. For holes, the Fermi-contact hyperfine interaction is strongly suppressed due to the predominantly p-like valence-band Bloch functions, and the remaining spin dephasing can involve non-contact hyperfine interaction, heavy-hole--light-hole mixing, charge-noise-induced fluctuations of the hole g-factor, and excitation-induced perturbations.

We therefore use Eq. (3) as an empirical fit function to extract an effective decay time of the observed DOCP oscillations, rather than as a microscopic model that assumes a purely Gaussian Overhauser-field distribution. The observation that alpha remains close to unity and varies with excitation power is indeed consistent with the presence of additional dephasing channels beyond a static Gaussian nuclear bath. In particular, the strong power dependence of both tau and alpha in the cw measurement suggests that optical re-excitation and excitation-induced perturbations contribute to the decay envelope.

For the pulsed measurement, alpha was fixed to unity to avoid overparameterization of the fit, since the number of delay points is limited and the main quantity of interest is the characteristic free-induction decay time. We have now clarified in the manuscript that alpha=1 is a pragmatic fitting choice and does not imply a microscopic exponential-noise model. We have also expanded the discussion of the extracted alpha values and their deviation from the Gaussian Overhauser-field limit for electrons.

4- The power dependence shown in Fig.2d is fitted as an exponential decay but this decay of dephasing time at high power is likely due to the shortening of the lifetime in the ground state (due to faster excitation) for which a simple model would likely give a dephasing time proportional to 1/(1/T2* + eta P^0.5) where P is the power and eta a fitting parameter.

Author response: Following the reviewer's suggestion, we reanalyzed the power dependence using the measurements presented in the revised Fig. 2d. We replaced the empirical exponential fit by a model in which the dephasing rate increases with excitation power. The phenomenological sqrt(P) model suggested by the reviewer, T2* (P) = 1/(1/T2* + eta P^0.5), provides a reasonable description of the data. However, the data are better described by an inverse-linear dependence, T2*(P) = 1/(m*P + b), corresponding to an approximately linear increase of the dephasing rate 1/T2* over the investigated power range. This behavior is consistent with an additional excitation-induced decoherence channel. A possible microscopic origin is an additional excitation event occurring before completion of the free spin evolution, effectively shortening the available ground-state evolution time and thereby reducing the measured T2*.

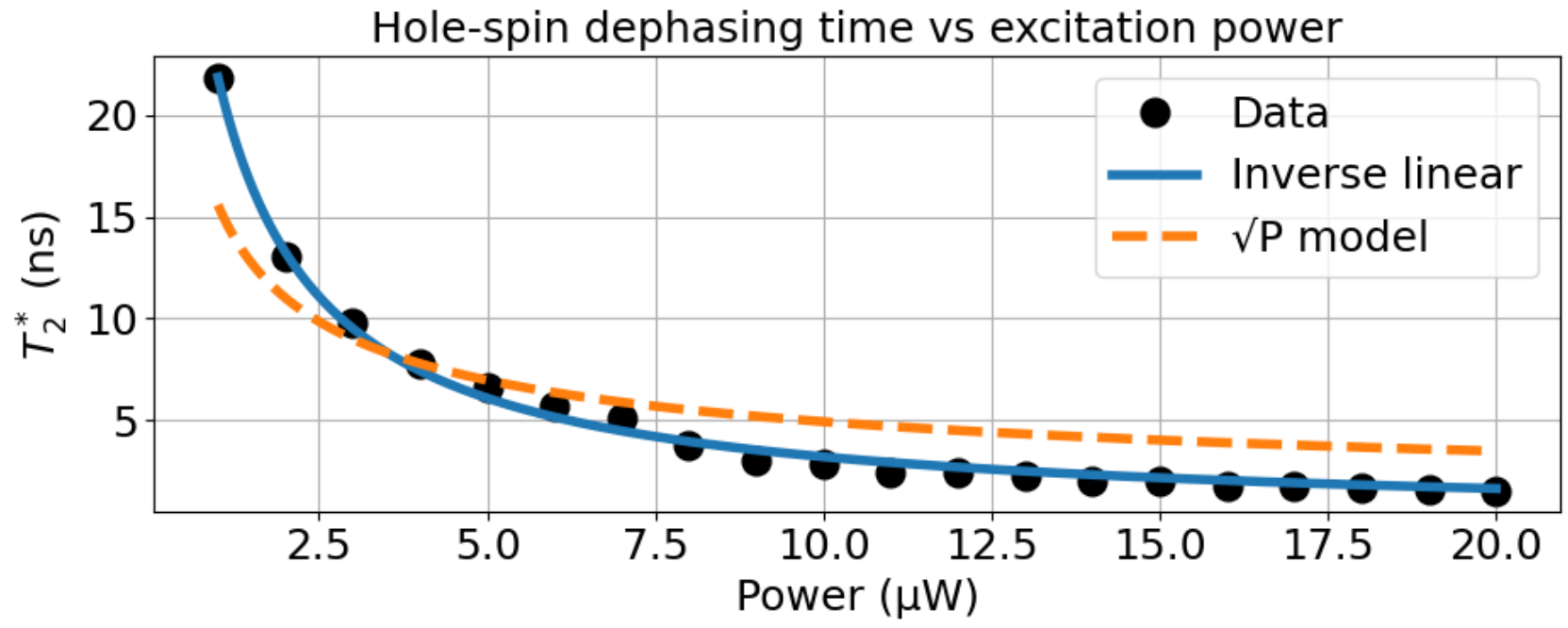

For completeness, corresponding power-dependent measurements obtained under circularly polarized excitation are retained in the Supplementary Information (Fig. S11), while the revised main text presents the more extensive dataset used for the quantitative analysis.

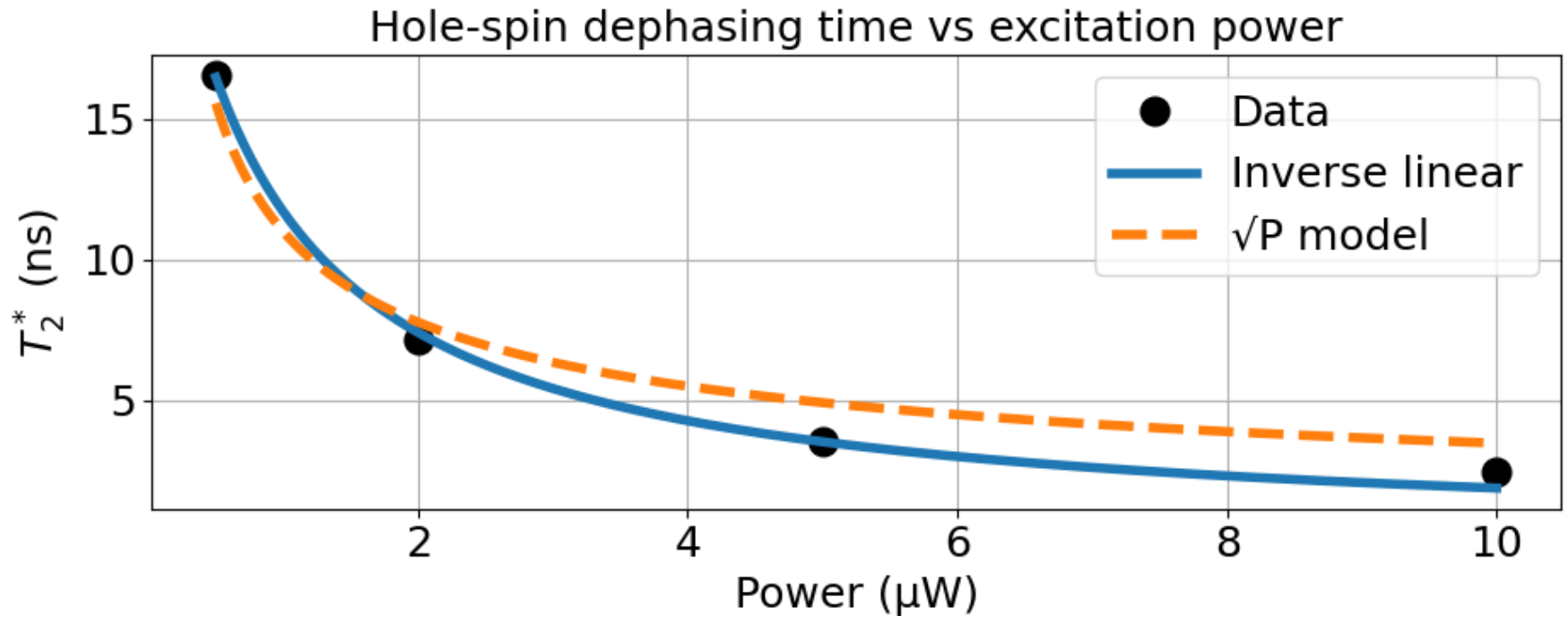

Accordingly, we revised the manuscript to emphasize an excitation-induced dephasing-rate contribution rather than an empirical exponential decay of T2*.

Manuscript: A Spin-Photon Interface in the Telecom C-Band with Long Hole Spin Dephasing Time

REVISION 2 / FINAL REVISION

REVIEWER COMMENTS

Reviewer #1 (Remarks to the Author):

The revised manuscript "A Spin-Photon Interface in the Telecom C-Band with Long Hole Spin Dephasing Time" by J. M. Michl et al. has improved significantly.

All my comments have been addressed, and I appreciate the detailed added discussions. In Particular, the point raised by reviewer 3 about the possible creation of unwanted DNP due to the circular polarization was very important.

For me the manuscript gives enough insights and advances our understanding that another smoking gun experiment is not needed. But I can understand if other reviewers see this differently.

Author response: We thank reviewer 1 for appreciating the improvements in our manuscript and for providing valuable feedback on the first revision round that led to an improved version of our manuscript.

Reviewer #2 (Remarks to the Author):

The authors have satisfactorily addressed all of my comments. The manuscript has been substantially improved in clarity and overall quality. I therefore recommend the manuscript for publication in Nature Communications.

Author response: We also thank reviewer 2 for the valuable feedback on our initial submission and for the positive response to our revisions.

Reviewer #3 (Remarks to the Author):

The authors have satisfactorily taken into account the criticisms I raised in my first review, notably by performing new measurements with different configuration of excitation polarization, and by extracting from their data the trion (i.e. electron) spin coherence of 1.2 ns. The main result of this study regarding the hole spin coherence of about 20ns in telecom QDs is quite convincing. Even though in my opinion this result is not really surprising, this type of research on telecom QDs is topical and the correlation-based measurements nicely illustrate the maturity of the technology which turns out very encouraging for future demonstration of multi-entangled photons at telecom wavelength. I therefore recommend publication in Nature Communication after a few (not totally minor) corrections/revisions.

We thank reviewer 3 for their critical and constructive feedback, which led to an improved version of our manuscript. Following we address the reviewer's comments on a point-by-point basis.

1- The abstract does not reflect an important conclusion of the study that the spin coherence time depends significantly on the magnetic field in the 0-150 mT range, and that the measurements with pulsed excitation need to be extended to lower magnetic fields to confirm this trend.

Author response: We have modified the last sentence of the abstract to read: *"We quantify the inhomogeneous dephasing times for the electron and hole for various magnetic fields and find a value of $T2^*$ = (21.8 ± 0.2) ns for the hole spin, for an optimal magnetic field of B = 20 mT. "*

The sentence now clearly states that we explored the dependency of the spin coherence on the strength of the external magnetic field. Regarding the reviewer's suggestion to extend the pulsed measurements to lower magnetic fields, we agree that this would be an interesting study. We believe these measurements represent future work rather than a conclusion of the present work and therefore we didn't include it in the abstract.

2- In fig 1b, the color code for H/V transitions should be swapped to be consistent with results in fig 1a.

Author response: We thank the reviewer for spotting this inconsistency. We have changed the colors in subplot 1b accordingly.

3- l.243 : the claim that pulse 1 initializes the spin is misleading... This is the detection in R or L polarization of the photon emitted after pulse 1 which effectively performs a projective measurement, and thus a kind of initialization. (As for the measurements in cw regime, the author could have used any linear polarization for pulse 1, which would suppress the oscillations along the vertical axis in fig 3b, while keeping the oscillations along the horizontal axis).

Author response: We agree with the reviewer about the physics. We thank her/him for pointing out that the description was not very precise and could be misinterpreted. To correctly describe the experiment, we thus have exchanged "pulse 1" with "the projection of the first photon" in the manuscript line referenced by the reviewer.

4- Using correlations of photons emitted under pulse excitation is not really a "novel approach" to measure the $T2^*$, since to some extent, it corresponds to a simpler version of the Lindner-Rudolph protocol which has been implemented by several teams to demonstrate cluster states with NIR quantum dots (e.g. Ref. 20 and 21 or more specifically Coste et al., Nat. Photonics 17, 582 (2023))). The authors should moderate the corresponding claim when they introduce the technique as well as in the conclusion.

Author response: We have removed the word "novel" at the beginning of the Results section and have replaced the words "novelty factor" in the Discussion with "point of interest".

5- There is no information about the time resolution/jitter of the detectors. This likely explains the reduced contrast of DOCP oscillations in fig.1 as compared to the spin conservation fidelity in zero field.

Author response: In a past publication (DOI: https://doi.org/10.1103/3qh7-b696) we have measured the response time uncertainty of our measurement setup to ~28 ps.

Fitting the instrument response function (IRF) from one of the datasets in Fig. 1d produced a value of 34 ps. With the given electron g-factor of ~2.1, the precession period for a magnetic field of 150 mT is ~227 ps, which is 7 times larger than the fitted IRF. While the finite temporal resolution is therefore expected to contribute to the reduced contrast at higher magnetic fields, it is unlikely to account for the observed reduction in the presented dataset.

Revision to the manuscript: no revisions.

6- While I agree that the measurements reported in Fig. S6 show no sign of DNP (the period of electron spin precession seems to be exactly the same in both configurations), the observation of a strong increase of DOCP under cw circular excitation reported in Fig. S10b could be in contrast interpreted as a reduction of the electron spin precession frequency due to DNP. The explanation that this increase is due to "deterministic re-initialization of the trion state in its |T↑⟩ (|T↓⟩) state through the L(R) polarized laser" sounds to me quite incorrect. The dependence of initial DOCP (namely just after hole spin initialization) on magnetic field is mostly governed by the trion spin precession during the lifetime (Hanle effect). Under H-polarized excitation it shows a Lorentzian-like decay above 10mT (as expected when there is no DNP), while under circular polarization the DOCP remains almost constant at a very high level as if the magnetic field experienced by the electron spin is strongly reduced due to DNP effect. The authors should be more careful when commenting the results of Fig. S10.

Author response: We thank the reviewer for this insight and agree that, based on the present data shown in Fig. S10b, it cannot be excluded that the electron spin experiences a reduced magnetic field due to DNP. As suggested by the reviewer, we have therefore revised the discussion in Supplementary Section S3.2 to a more careful phrasing. We conclude our findings now with the sentence: *"Whether this effect arises solely from spin-selective initialization governed by the trion polarization selection rules or instead reflects dynamic nuclear polarization induced by continuous circularly polarized excitation remains inconclusive at this point."*

We would also like to highlight that, as the reviewer has observed above, the initial DOCP for linear (H-polarized) excitation shows a dependency on magnetic field that is in line with the expectations when there is no DNP. This further solidifies our findings in the main manuscript (Figure 2) where we show results for the hole spin obtained using linear excitation polarization. We want to explicitly express our gratitude towards reviewer 3 again at this point for their remarks during the first round of review. There, the reviewer brought our attention to the importance of using linear polarization which led to the acquisition of new datasets, significantly strengthening our manuscript.

While an additional study of the impact of DNP on electron trion state spin precession is generally of interest, it is beyond the scope of this manuscript which focuses on the dynamics of a ground-state hole spin.
